# *DeepDynaForecast*: Phylogenetic-informed graph deep learning for epidemic transmission dynamic prediction

**DOI:** 10.1371/journal.pcbi.1011351

**Published:** 2024-04-10

**Authors:** Chaoyue Sun, Ruogu Fang, Marco Salemi, Mattia Prosperi, Brittany Rife Magalis

**Affiliations:** 1 Department of Electrical and Computer Engineering, Herbert Wertheim College of Engineering, University of Florida, Gainesville, Florida, United States of America; 2 J. Crayton Pruitt Family Department of Biomedical Engineering, Herbert Wertheim College of Engineering, University of Florida, Gainesville, Florida, United States of America; 3 Center for Cognitive Aging and Memory, McKnight Brain Institute, University of Florida, Gainesville, Florida, United States of America; 4 Department of Pathology, Immunology, and Laboratory Medicine, University of Florida, Gainesville, Florida, United States of America; 5 Emerging Pathogens Institute, University of Florida, Gainesville, Florida, United States of America; 6 Department of Epidemiology, University of Florida, Gainesville, Florida, United States of America; ETH Zurich, SWITZERLAND

## Abstract

In the midst of an outbreak or sustained epidemic, reliable prediction of transmission risks and patterns of spread is critical to inform public health programs. Projections of transmission growth or decline among specific risk groups can aid in optimizing interventions, particularly when resources are limited. Phylogenetic trees have been widely used in the detection of transmission chains and high-risk populations. Moreover, tree topology and the incorporation of population parameters (phylodynamics) can be useful in reconstructing the evolutionary dynamics of an epidemic across space and time among individuals. We now demonstrate the utility of phylodynamic trees for transmission modeling and forecasting, developing a phylogeny-based deep learning system, referred to as *DeepDynaForecast*. Our approach leverages a primal-dual graph learning structure with shortcut multi-layer aggregation, which is suited for the early identification and prediction of transmission dynamics in emerging high-risk groups. We demonstrate the accuracy of *DeepDynaForecast* using simulated outbreak data and the utility of the learned model using empirical, large-scale data from the human immunodeficiency virus epidemic in Florida between 2012 and 2020. Our framework is available as open-source software (MIT license) at github.com/lab-smile/DeepDynaForcast.

## Author summary

During an outbreak or sustained epidemic, accurate prediction of patterns in transmission risk can reliably inform public health strategies. Projections indicating growth or decline of transmission for specific risk groups can significantly enhance the optimization of interventions, especially when resources are limited. To address this, we present *DeepDynaForecast*, a cutting-edge deep learning algorithm designed for forecasting pathogen transmission dynamics. Uniquely, *DeepDynaForecast* was trained on in-depth simulation data, classifying samples according to their dynamics (growth, static, or decline) with accuracy of 91.6%. We evaluated the model’s performance and application using simulated outbreak data and empirical, large-scale data from the HIV epidemic in Florida between 2012 and 2020. We conclude *DeepDynaForecast* represents a significant advancement in genomics-mediated pathogen transmission characterization and has the potential to catalyze new research directions within virology, molecular biology, and public health.

## Introduction

Epidemiological modeling of the spread of a disease during an outbreak is critical in predicting the devastation caused by the root pathogen and in designing specific public health interventions to curb unfavorable outcomes. The vast majority of the models used, however, often assume random mixing of the host population, which can affect parameter estimates used in these predictions (e.g., [1–4]). Awareness of this limitation often exists, but incorporation of the numerous relevant structures within a population requires *a priori* information regarding not only population behavior but also transmission routes of the pathogen responsible for the outbreak. For example, increased transmission of food-borne pathogens may be found among individuals primarily relying on a local food vendor [5]. Moreover, the population structure may be dynamic, as in the case of brief social gatherings wherein airborne pathogens are more easily spread. Groups of infected individuals for which risk of pathogen transmission is heightened, regardless of their episodic nature, can be identified through case investigation and contact tracing. The resources required in this endeavor are often not available or difficult to expand in the time required during unexpected public health emergencies. Combined pathogen molecular data and patient information can be used to identify these groups, thereby narrowing the scope of contact tracing efforts and providing invaluable insight into patterns of spread for which targeted interventions can aid in curbing an outbreak.

Pathogen genomic data have become increasingly used in outbreak surveillance primarily owing to the evolutionary information they provide for the development of therapeutic interventions, a primary recent example being SARS-CoV-2 [6]. The evolutionary trajectory of a pathogen can be readily monitored owing to the rapid accumulation of mutations that result from the short generation time and/or infidelity of the replication machinery characteristic of micro-organisms. The rate of accumulation of mutations, particularly for viruses [7], is often deterministic and so is proportional to the number of replication cycles. Assuming the replication rate is relatively constant for a particular pathogen during infection, the number of replication cycles, and thus mutations, can be estimated from the time of infection of one individual and time of transmission from that individual to another. Fewer mutations are thus expected to occur for shorter transmission times when transmission is more likely. This relationship of evolution and transmission is the principle for genetic clustering, wherein for a population sample, transmission clusters are defined as groups of patient-derived pathogen sequences characterized by minimal genetic variation, representing captured transmission events.

Genetic clustering can be achieved using a variety of algorithms. Primarily distance-based methods [8, 9] rely on a user-specified threshold during pairwise genetic distance estimation among sequences, below which sequences are considered to be connected. These methods are fast and accurate, but not without limitations. Firstly, application is limited when *a priori* mutation information required for threshold specification is unknown. I.e., a threshold of three mutational differences can be set when prior contact tracing and sampling have demonstrated at most three mutational differences between known transmission pairs. The use of deep learning has been proposed by Kupperman *et. al*. [10] to overcome this limitation. However, the resolution of these clustering-based methods, regardless of the need for threshold specification, is limited to the level of the group, as specific mutation information is not used to resolve individual relationships within or among groups. Alternatively, a phylogenetic tree reconstructed from the set of mutational information at genomic sites offers increased resolution for individual relationships.

Another benefit to phylogenetic inference is that branches within the tree can adopt generalized shapes, or topological features, that can provide critical information as to the underlying transmission contact dynamics [11, 12] (e.g., presence of “super-spreaders”) and population dynamics over time [13]. Importantly, neighboring samples within a tree that share inferred contact dynamics (e.g., high transmission rate) may provide critical risk group information, removing the need for clustering algorithms entirely. This integration of population genetic parameterization with phylogenetics is known as “phylodynamics” and is based on retroactively tracing an epidemic backward in time. For this reason, phylodynamic methods have been largely applied to large populations, rather than for real-time monitoring of smaller risk groups during the early stages of an epidemic. There do exist phylogeny-based dynamic forecasting models, but they are 1) again assume a population structured into risk groups determined by clustering thresholds and 2) are limited to forecasting growth [9, 14–16]. Moreover, growth in these models is defined in terms of number of infected individuals, rather than as a function of transmission rate and so may not capture risk groups for which the transmission rate itself has recently increased, owing to, for example, transient behavioral changes. As a result, the question remains as to the *predictive* power of phylodynamic information for the characterization of different risk group transmission dynamics, particularly when supplied at the onset of an outbreak when samples are scarce.

Regardless of whether a distance-based or phylogeny-based cluster detection method is being used, focus has been traditionally placed on clusters based on size (e.g., [17–19]), represented by the number of sampled individuals included in the cluster. This phenomenon may be in part due to the Center for Disease Control (CDC) guidelines, which specify that, for human immunodeficiency virus (HIV) outbreaks in most jurisdictions, clusters of ≥ 5 individuals should be considered clusters of priority, or concern. Specifically, once the larger of clusters is identified, existing patient data (and additionally gathered data if necessary) are to be used to identify which persons are out of care or lack viral suppression, for which targeted interventions should be employed [20]. Hence, prioritization has been given to larger clusters, despite acknowledgment of sampling bias and the lack of evidence linking cluster size to transmission potential [21]. Moreover, a clear definition for what should be considered “larger” clusters has not been established and is up to the researcher to decide.

**Fig 1:**
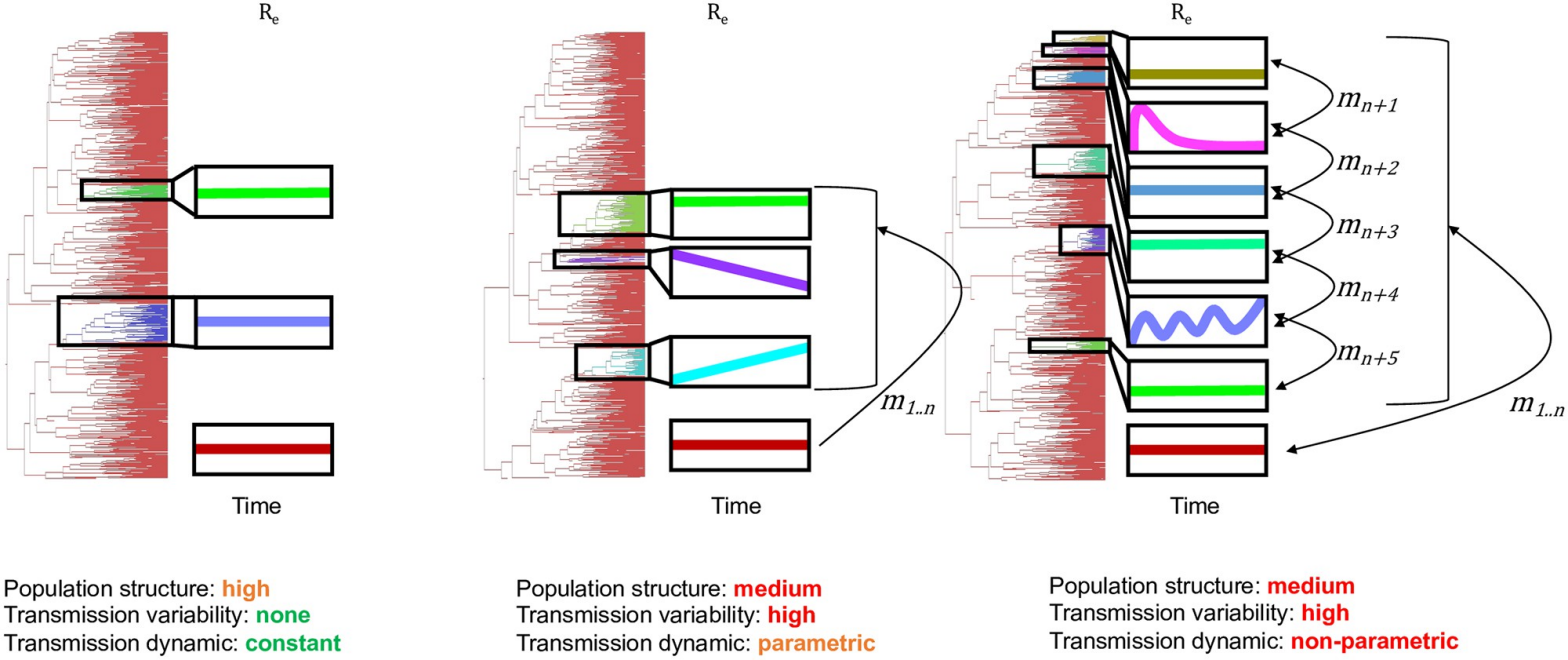
Ranging complexity of tree topological features resulting from a structured infected population. *n* transmission clusters, distinct from the background population (maroon), can vary in effective reproductive number (*R*_*e*_) over time (x-axis) and rate of infection (*m*) by or to individuals from other groups. Variations in transmission dynamics are imprinted in branch patterns within the corresponding phylogeny and can aid in identifying groups of interest.

In recent years, deep learning has been applied extensively in epidemiology, allowing for increased information gained from large datasets comprising numerous multi-factorial risk factors of infection and spread [22, 23]. Neural networks have also gained popularity among sequencing endeavors, used in identifying connections between mutations within high-dimensional genome sequence data and disease status [24]. Prior research has also explored the use of convolutional neural networks (CNNs) to predict infection outbreaks based on sequencing data [10]. However, this approach primarily focuses on identifying “active” or “inactive” individuals within risk groups and does not adequately capture individual relationships, nor the dynamics of transmission. The deep learning approach developed by Voznica *et. al*. [4] utilizes instead phylogenetic trees, but the focus is on global transmission parameters (e.g., *R*_0_), rather than dynamics among individual risk groups. To address these gaps, we developed the *DeepDynaForecast* graph neural network (GNN) model, designed to predict near-future transmission dynamics for individual samples within a phylogenetic tree. GNNs have been specifically designed for data that could be represented as a graph structure connected via complex relationships [25–27], for which the phylogenetic tree is a prime candidate. Complex relationships within outbreak phylogenies can range from simple community-based structures, with isolated risk groups transmitting at constant rates, to risk group mixing with varying, non-deterministic transmission rates between groups and over time (Fig 1). We explored this complexity by simulating phylogenies representing viral and bacterial outbreaks at varying times during the epidemic and comprised of smaller risk groups with deterministic, yet varying transmission rates relative to the background population. Using these data, we demonstrate the effectiveness of our GNN method (*DeepDynaForecast*) in predicting future dynamics from limited, early sampling and its promising capacity to improve public health efforts in preventing epidemic spread. We then sought to apply the trained model to the HIV epidemic in Florida between 2012 and 2020, for which large-scale pathogen sequencing data and metadata is available, previously analyzed using traditional distance-based methods [19].

## Results

### *DeepDynaForecast* framework

**Fig 2:**
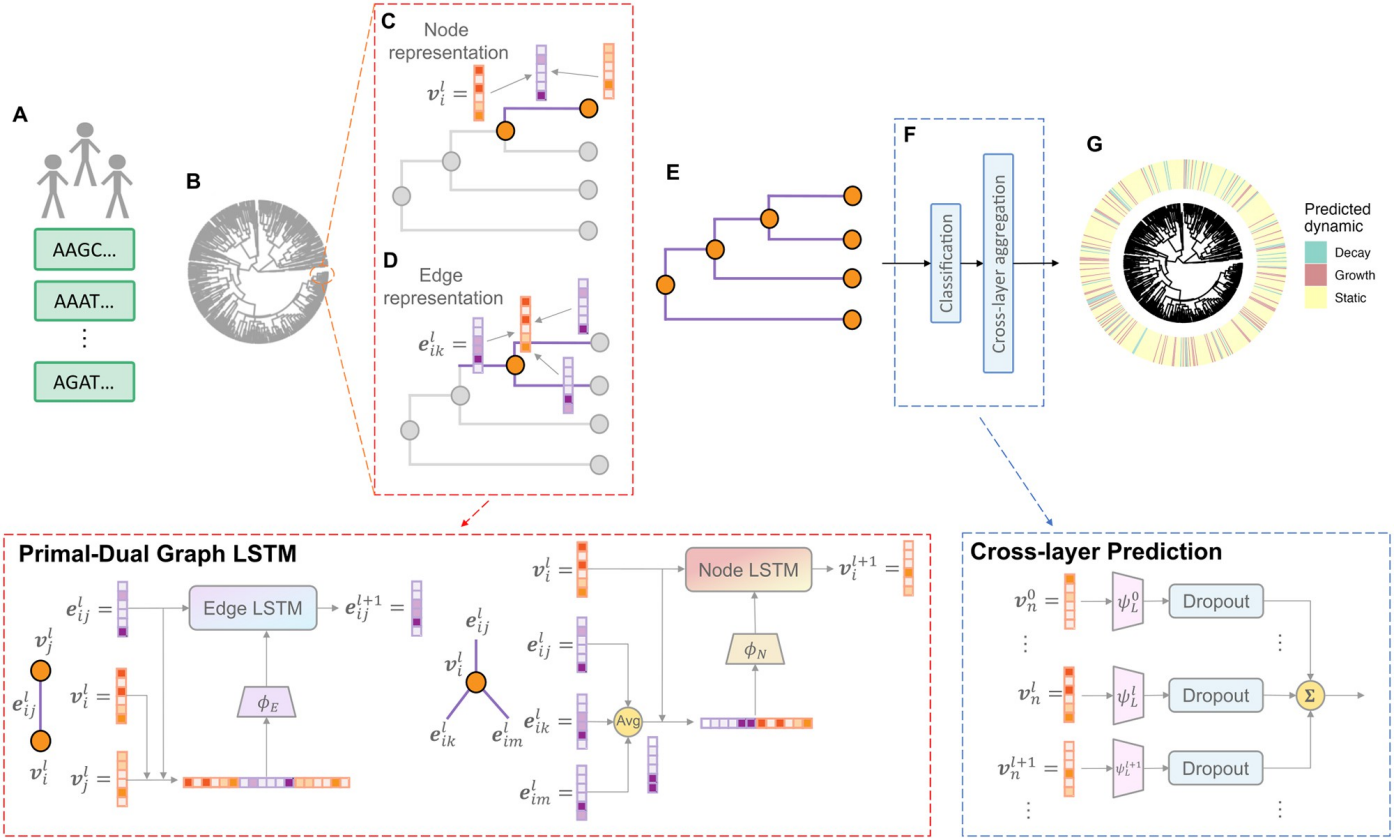
Our proposed *DeepDynaForecast* architecture. A: Pathogen genomic data collected during outbreak molecular surveillance. B: A phylogenetic tree, reconstructed from the genomic data, which is used as input to trace the transmission among the populations. In this tree, nodes represent individuals, while edges represented transmission or mutation events. The phylogenetic tree is modeled using a bi-directed graph, where initial node representation vector **v**_*i*_ is randomly generated for each node *i*, and edge representation vector **e**_*ij*_ for edge *e*_*ij*_ from node *i* to *j* is initialized from the branch length with a neural network. C-D: Example of Primal-Dual Graph Long Short-Term Memory (PDGLSTM) learning architecture on a subtree to update **v**_*i*_ and **e**_*ij*_ at the *l*-th layer. Two parallel LSTM modules are utilized to update the node and edge representations in each message-passing iteration. Within this process, each edge/node aggregates adjacent node/edge representations and encodes low-dimensional messages by the neural networks *ϕ*_*E*_ and *ϕ*_*N*_. These node/edge messages are input into their corresponding LSTM modules to facilitate the update of node and edge representations E: This system sequentially applies *N* rounds of message-passing iterations, thus producing updated nodes and edges representations. F: Cross-layer Prediction (CLP) module on each leaf node. A series of neural networks {*ψ*_*L*_} are engaged in predicting the dynamics of leaf *n* using various levels of node representations {**v**_*n*_}. This process is followed by dropout layers and summation operations to generate the final prediction. G: Predicted dynamics for leaves on the phylogenetic tree.

Our *DeepDynaForecast* model was developed to predict near-future transmission dynamics for individual samples within a phylogenetic tree, focusing on accurately classifying nodes as indicative of growing, decaying, or static transmission. Fig 2 illustrates the resulting computational framework. The framework utilizes a phylogenetic tree, reconstructed from pathogen genomic data collected during outbreak molecular surveillance. Subsequently, the *DeepDynaForecast* model harnesses two primary components: a primal-dual graph learning architecture and a cross-layer dynamics prediction module. The detailed methodology of the framework is provided in the Methods section, while the ensuing sections provide succinct introductions to each key component.

#### Primal-dual graph LSTM module (PDGLSTM)

The PDGLSTM [28] was developed in order to earn the node and edge representations simultaneously by applying two parallel GLSTMs on nodes and edges. GLSTM incorporates LSTM to learn about the sequential representations generated from multiple message-passing iterations. As shown in Fig 2, each edge/node aggregates neighboring node/edge representations and encodes low-dimensional messages by the neural networks *ϕ*_*E*_ and *ϕ*_*N*_. These node/edge messages are input into their corresponding LSTM modules to facilitate the update of node and edge representations.

#### Cross-layer Prediction module (CLP)

After sequentially *N* times of message-passing iterations, the CLP module was/is applied to predict the dynamics of leaves and masks internal nodes when predicting. A series of neural networks {*ψ*_*L*_} are engaged in predicting the dynamics of leaves using various levels of node representations {**v**}. This process is followed by dropout layers and summation operations to make the final result robust.

### *DeepDynaForecast* performance

DeepDynaForecast’s performance evaluation was based on the ability to predict the dynamic transmission status of individual nodes within ground truth phylogenies. Simulated phylogenies harbored risk groups categorized as exhibiting static transmission rates over time or linear trends in transmission rate growth or decline (see Methods and Supplementary S1 Table). Table 1 compares two baseline models—Graph Convolutional Network (GCN) [29] and Graph Isomorphism Network (GIN) [25]—and *DeepDynaForecast*-based models for three training scenarios: acute infectious respiratory virus (ARI) outbreak simulations (DDF_ARI), *Mycobacterium Tuberculosis* (TB) and HIV co-infection simulations (DDF_TB), and the combination of both dataset types (DDF_ARI_TB). Two baseline models were trained on the combined simulations, and all five models were evaluated for ARI, TB, and their combination (Table 1). The metrics included accuracy, F1-score, precision, area under the receiver operating characteristic (AUROC), Brier score (BS), and Cross-entropy (For further details, see performance evaluation in the Methods section).

**Table 1:** Performance for two baseline models and *DeepDynaForecast* on transmission dynamic prediction of external nodes with three training scenarios.

Model	Dataset	Accuracy ↑	F1 ↑	Precision ↑	AUROC ↑	BS ↓	CE ↓
GCN	ARI	0.640	0.298	0.370	0.753	0.444	0.729
	TB	0.556	0.240	0.350	0.676	0.537	0.899
	ARI+TB	0.612	0.274	0.362	0.737	0.470	0.775
GIN	ARI	0.668	0.308	0.377	0.767	0.421	0.696
	TB	0.585	0.284	0.354	0.710	0.528	0.894
	ARI+TB	0.654	0.299	0.368	0.763	0.442	0.736
DDF_ARI	ARI	0.909	0.539	0.473	0.975	0.140	0.264
	TB	0.687	0.257	0.379	0.875	0.492	0.948
	ARI+TB	0.823	0.380	0.399	0.930	0.277	0.525
DDF_TB	ARI	0.635	0.488	0.567	0.875	0.633	1.741
	TB	0.768	*0.461*	*0.419*	0.950	0.339	0.608
	ARI+TB	0.636	0.481	0.437	0.858	0.621	1.659
DDF_ARI_TB	ARI	0.931	0.545	0.477	0.982	0.108	0.200
	TB	*0.816*	0.433	0.401	*0.954*	*0.271*	*0.492*
	ARI+TB	**0.916**	**0.512**	**0.452**	**0.977**	**0.128**	**0.237**

Baseline models comprised Graph Convolutional Network (GCN) and Graph Isomorphism Network (GIN). The training scenarios included the model trained with Respiratory virus simulations only (DDF_ARI), the model trained on *Mycobacterium tuberculosis* simulations only (DDF_TB) and the model trained on the mixed type of transmission patterns (DDF_ARI_TB). Baseline models were also trained on the combination of ARI and TB. To mitigate the impact of unbalanced label distribution, metrics for multi-class node types—including accuracy, F1-score, precision, and area under the receiver operating characteristic (AUROC)—were uniformly aggregated across the three classes. Weighted Brier score (BS) and weighted Cross-entropy (CE) were calculated based on predicted probabilities adjusted by the inverse prevalence of classes, providing “soft” evaluations of the models. For each testing dataset, models were assessed on ARI trees, TB trees, and a combination of both. The best performance for each evaluation metric among the five models is highlighted using underlining, italics, and bold for ARI, TB, and the combination, respectively.

For model performance using combined ARI and TB datasets, *DeepDynaForecast* achieved 91.6% balanced accuracy and 0.977 macro AUROC, outperforming the best baseline model (GIN) by 40.1% and 28.0%. Another significant improvement from GIN to DDF_ARI_TB is observed in the “soft” evaluations of the model. For example, DDF_ARI_TB reduces the weighted Brier score by 71.0% (from 0.442 to 0.128) and shows a similar trend for weight Cross-entropy (from 0.736 to 0.237). Similar conclusions could be drawn considering all six metrics on either ARI testing or TB testing with GCN, GIN, and DDF_ARI_TB. This analysis demonstrates that the *DeepDynaForecast* architecture can learn more comprehensive transmission patterns compared to GCN and GIN.

To explore the impact of different training scenarios, we compared *DeepDynaForecast*’s performance when trained on ARI only, TB only, and their combination. When tested on combined ARI and TB datasets, DDF_ARI_TB improved balanced accuracy by 11.3% over DDF_ARI and 44.0% over DDF_TB. For all six metrics, DDF_ARI_TB consistently outperformed DDF_ARI and DDF_TB. Considering DDF_ARI_TB was trained on a larger dataset compared to DDF_ARI and DDF_TB, we explored how the size of the training data and the diversity of training scenarios contributed to model performance. As detailed in Supplementary S6 Table, for the ARI data, we found that increasing the dataset size was as effective as generalizing the training scenario. In contrast, for the TB data, an increase in dataset size proved more beneficial. These observations imply that beyond the advantages gained from larger training sets, DDF_ARI_TB also benefits from the varied nature of its training scenarios. Notably, even in evaluations focused solely on ARI, DDF_ARI_TB surpassed DDF_ARI in five of the six metrics, further supporting the generalization capabilities of a model trained on mixed transmission types. In terms of performance on TB, although TB trees were not part of DDF_ARI’s training, it still achieved a 68.7% balanced accuracy and a 0.875 AUROC, with only a 15.8% and 7.9% decrease in balanced accuracy and AUROC, respectively, compared to DDF_ARI_TB. These findings indicate that *DeepDynaForecast* can be used for both acute and chronic pathogen transmission patterns and excels in learning mixed transmission patterns.

While the TB-only model (DDF_TB) performed well when evaluated exclusively on TB data (attaining a balanced accuracy of 76.8% and a macro AUROC of 0.950), it fell short in predicting dynamic status for ARI nodes. This discrepancy suggests that the branch patterns characteristic of chronic infection epidemics, such as TB, are distinct from those associated with acute infections. The observed performance deficit could be ascribed to the reduced representation of TB decaying transmission nodes (51,419) compared to those of ARI (589,119)—for more details on node representation, refer to the Methods section. This observation further elucidates why the GCN, GIN, and DDF_ARI_TB models yield less effective performance on TB testing than ARI testing when trained in the combination of ARI and TB.

**Fig 3:**
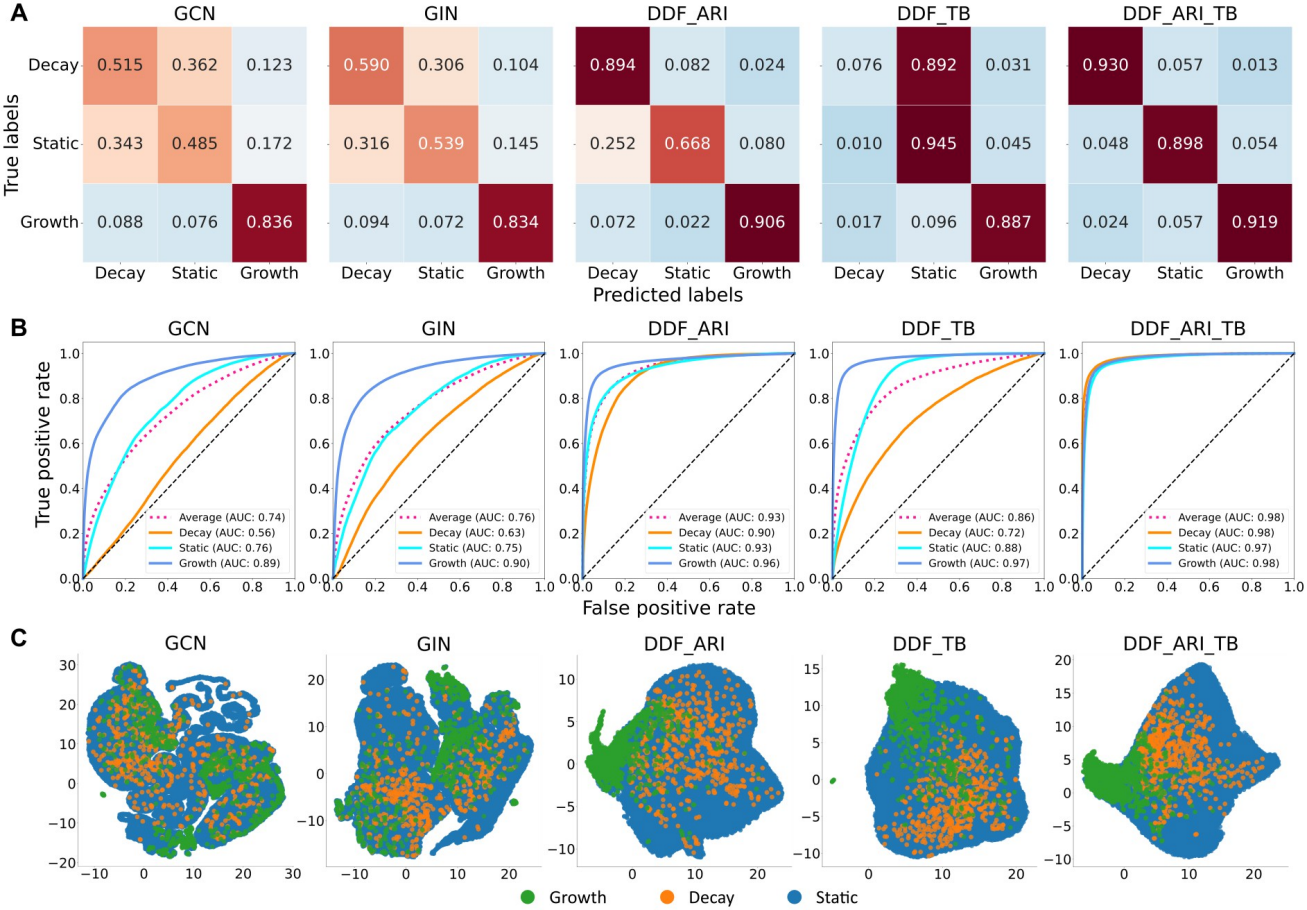
Figurative performance comparison of five models on combined ARI and TB test sets. A: Confusion matrices with row-wise normalized elements. B: One-verse-rest receiver operator characteristic curve (ROC) for each class and a macro averaged ROC curve with magenta dash lines. The corresponding AUCs are indicated for each curve. C: UMAP visualization of aggregation of learned node representations in message-passing iterations. Plots were generated in randomly sampled 50 phylogenetic trees in ARI and TB test sets.

In addition to comparing method performance for specific datasets, we also utilized the resulting confusion matrices to summarize and illustrate detailed prediction distributions across different risk group types. Fig 3A displays the confusion matrices of various approaches, with each element normalized by the number of true labels summarized over each row for intuitive comparison. Consistent with the conclusions drawn from the metrics, DDF_ARI_TB achieved 93.0%, 89.8%, and 91.9% accuracy on nodes representing decaying, static, and growing transmission rate groups, respectively, outperforming all five models. Decay and static leaves within the tree were often misclassified in GCN, GIN, and DDF_TB, but this issue was mitigated in DDF_ARI and DDF_ARI_TB. Fig 3B demonstrates a one-versus-rest receiver operator characteristic curve (ROC) for each class and a macro-averaged ROC curve. DDF_ARI_TB attained > 0.96 AUROC for all four curves, exhibiting the best performance. Fig 3C represents the UMAP [30] 2-dimensional (2D) feature space generated from an aggregation of learned node representations. Decaying transmission nodes were less clustered in the UMAP space than growth samples, explaining why static and decaying transmission samples were more prone to misclassification. As expected, nodes with the same labels were better clustered in DDF approaches.

### Model limitation analysis

**Fig 4:**
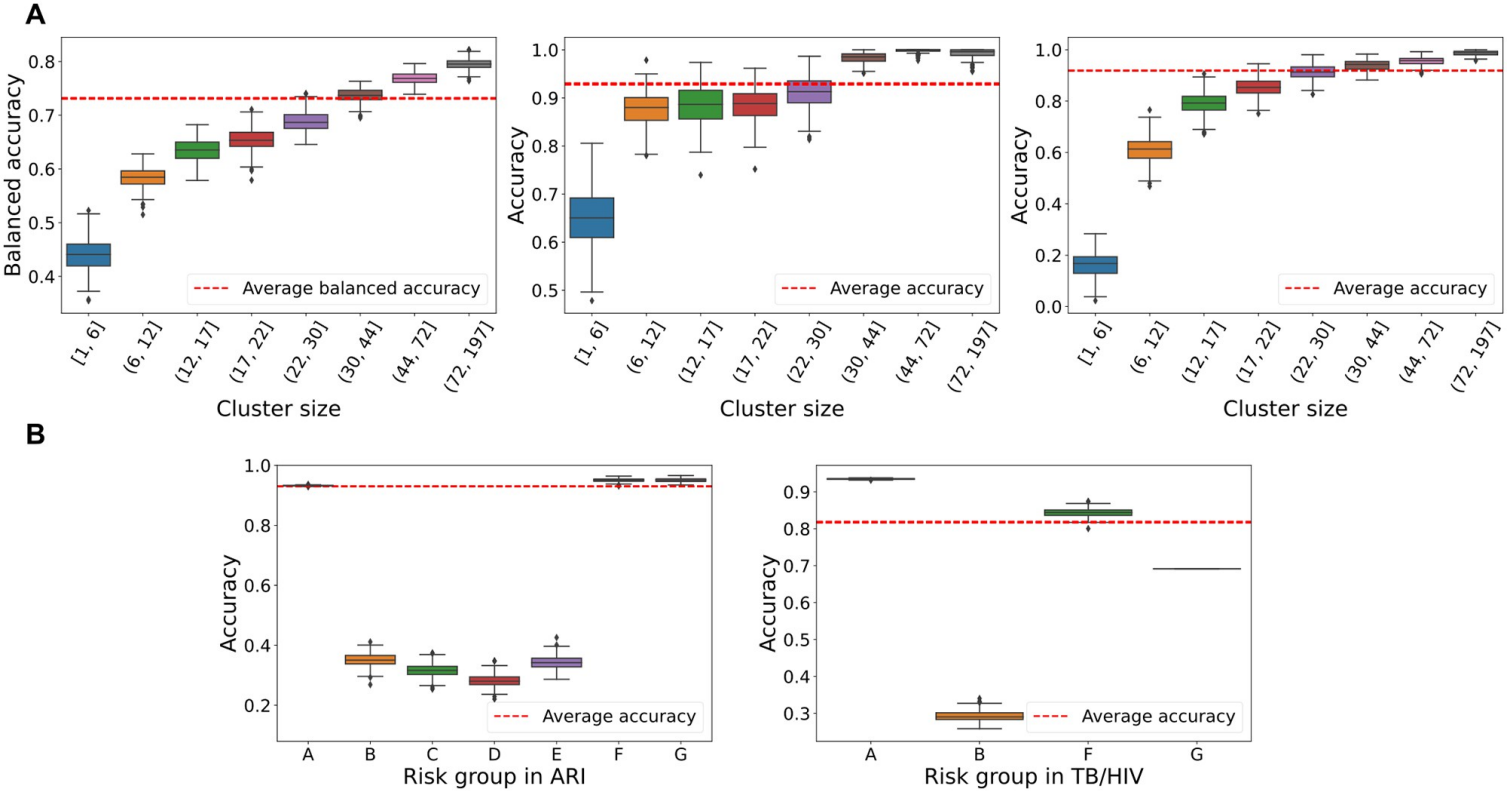
Sensitivity of the DeepDynaForecast model to cluster size and risk group transmission type. A: Balanced accuracy when predicting leaves within groups of different external node sizes. Binned intervals for quantitative data were generated using eight quantiles. Left panel: model performance among all groups. Middle panel: model performance on decaying clusters only. Right panel: model performance on growing clusters only. B: Performance of varying risk groups in ARI and TB simulations (see Supplementary S1 Table).

In order to assess the limitations of the *DeepDynaForecast* model for future application to structured datasets, we conducted a limitation analysis to determine the prediction accuracy distribution over group size for each transmission dynamic category, wherein group size is represented by the number of leaves within the phylogeny. The results demonstrate a consistent reduction in accuracy across all risk groups with reduced group sizes. Specifically, the *DeepDynaForecast* model requires a sample size ≥ 30 individuals to achieve the proposed level of accuracy across all three types of transmission dynamic (Fig 4A, left panel). We observed that, while prediction of transmission growth was less sensitive to cluster size than prediction of decay (≥ 20), accuracy dropped remarkably (< 20%) for sizes ≤ 6 (Fig 4A, right panel). Conversely, for decaying nodes, accuracy remained around 90% for group sizes > 6 and still exceeded 60% in clusters of size ≤ 6 or smaller (Fig 4A, middle panel), indicating a relative robustness in prediction accuracy across different cluster sizes for prediction of transmission decline.

Furthermore, we evaluated the model’s performance for individual risk group types, representing variations of the three categories of time-dependent transmission—static, growth, or decay. Variations were derived from pre-defined transmission dynamic parameters during data simulation (Supplementary S1 Table). In addition to linear growth and decline in transmission rate (groups F and G), rates among static groups were allowed to vary by group (ARI groups A-D, TB groups A-B) in order to determine the ability of the model to distinguish high-rate static transmission from growing transmission over time and, conversely, low-rate static transmission from decay. For the simulations, background nodes (nodes not assigned to risk groups), which represented the majority of the infected population, were considered group A and also exhibited static transmission. The model achieved optimal performance for groups A, F, and G for ARI simulations but exhibited reduced performance for static transmission risk groups with rates varying from the background population (Fig 4B, left panel, and Supplementary S4 Table). Evaluation of the TB/HIV co-infection data presented with similar conclusions (Fig 4B, right panel), with optimal performance for groups A (background) and F (growth) but poor performance for group B (static risk group), suggesting difficulties in distinguishing high-rate transmission from transmission rate growth and vice versa, regardless of infection type (chronic, acute). Additionally, relatively poorer performance was observed for group E (decay), consistent with the findings of the confusion matrix, though not below (70%).

### Transmission dynamics of the HIV epidemic in Florida

**Fig 5:**
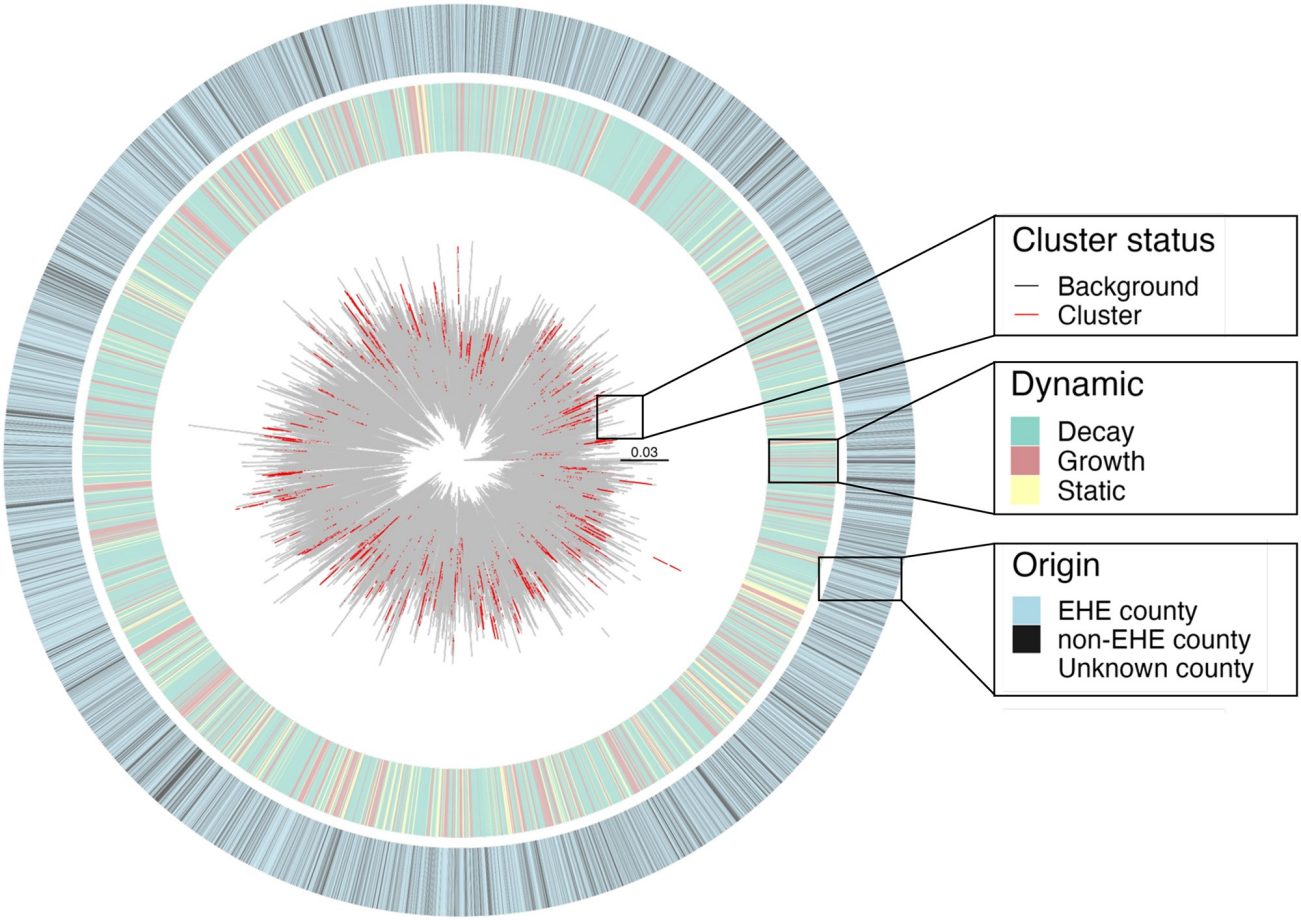
Florida HIV-1 subtype B *pol* sequence phylogeny (2012–2017). The maximum likelihood phylogeny was generated as described in Rich *et al*. [19] for 27, 115 partial *pol* sequences sampled from individuals across the state of Florida, for whom metadata were provided. County of residence, categorized according to EHE prioritization, is shown in the corresponding heatmap. Cluster status for each external branch according to MicrobeTrace [33] and dynamic prediction using the *DeepDynaForecast* are also shown. Branches are scaled in substitutions/site.

We next sought to apply the trained model to an HIV outbreak, for which both sequence and patient data were available and for which distinct transmission dynamics were hypothesized to be represented in the phylogeny. Persons living with HIV (PLWH), in general, are an ideal example of a structured population with disproportionate transmission patterns across geographical regions and risk groups [31]. In 2017, rates of new HIV diagnoses in the United States (U.S.) were highest in the southern region, where the state of Florida in particular exhibited the highest number of new diagnoses [31]. In 2019, the U.S. Department of Health and Human Services released the federal plan for Ending the HIV Epidemic (EHE) within 10 years, identifying 48 counties with high incidences of HIV diagnoses, including seven urban Florida counties as high-priority areas. Strategic interventions for these areas largely revolved around building the capacity to detect and respond to ongoing and emerging clusters [32], dependent on molecular epidemiology techniques. The Florida Department of Health (FDOH) has been actively collecting partial HIV-1 polymerase (*pol*) genetic sequences from surveillance laboratories since 2007 to attain an analyzable HIV nucleotide sequence within 12 months of diagnosis for >60% of persons diagnosed with HIV per year, culminating in a total of 28, 098 partial HIV-1 *pol* sequences. Of these, 27, 115 (96.5%) were classified as subtype B and used in previous clustering analysis by Rich *et al*. [19]. In the Rich *et al*. study, MicrobeTrace [33] was used to identify potential clusters of transmission. For this clustering approach, a pairwise genetic distance of 1.5% was used as a threshold, below which individuals were considered to be related via transmission chain or high-risk transmission group (Fig 5). Larger transmission clusters, spanning 11–70 individuals, were prioritized and assessed for putative risk factors using patient metadata provided from the FDOH. These data included year of HIV genotype determination, year of birth, birth region, gender at birth, race/ethnicity, county of residence, and mode of transmission exposure. In this previous study, cluster size was inversely associated with the age of cluster members, with a greater prevalence of younger PLWH detected in the larger, prioritized clusters. Owing to the uncertainty surrounding the 1) threshold values required for cluster identification and 2) cluster size cutoffs required to define larger clusters, we sought to demonstrate the utility of *DeepDynaForecast* as an alternative, cluster-agnostic tool that can clearly define transmission dynamics with anticipated risk group associations (in this case, age).

**Fig 6:**
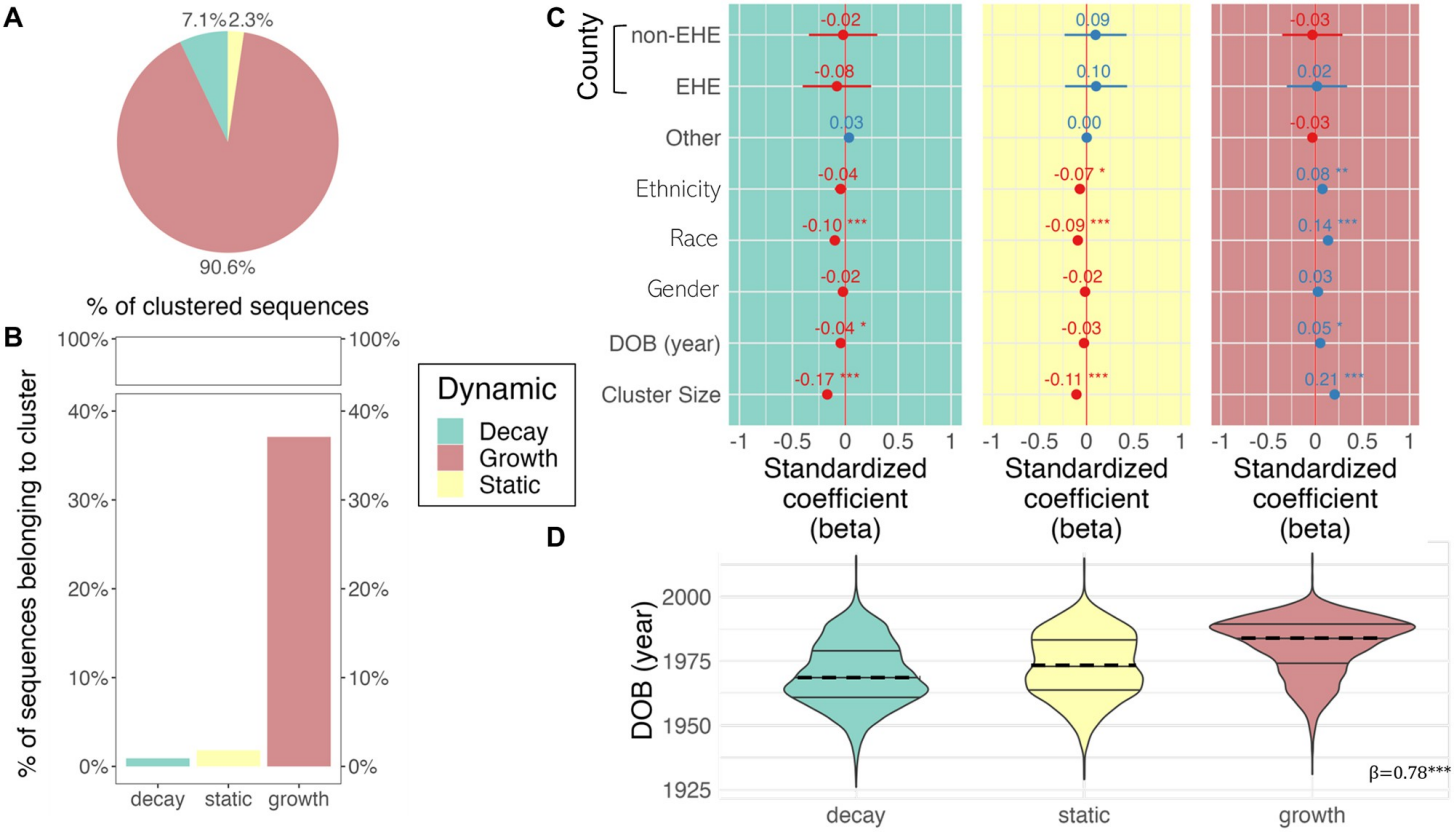
Relationship of predicted transmission dynamics with genetic clustering and patient risk factors. Transmission clusters were previously identified in Rich *et al*. [19] using MicrobeTrace [33] and the corresponding information used herein to determine the percentage of clustered sequences classified according to prediction category (A), as well as the percentage of predicted categories represented among clustered sequences (B). A multivariate logistic regression model was used to identify predictors for each transmission category, regardless of clustering status (C). Only risk factor categories with significant or marginally significant predictors are depicted, with the exception of the inclusion of county of origin, owing to its importance in clustering [34]. A linear regression model, treating prediction categories as ordinal, was used to quantify the relationship between year of birth of individuals and prediction status (D). Prediction categories refer to growth, decay, or static transmission relative to the background, or majority, infected population and were determined using the trained PGLSTM model implemented in *DeepDynaForecast*.

When *DeepDynaForecast* was applied to the Florida outbreak, ≤ 40% of individuals were classified as predictive of future growth or decline in transmission. It is important to note that, similar to clustering methods, *DeepDynaForecast* assumes the majority of sampled individuals within an infected population transmit at a constant, or static, rate, with a minority of individuals demonstrating transmission growth or decay, relative to the majority (“background”) population. In other words, *DeepDynaForecast* was trained on outbreak scenarios wherein dynamic risk groups comprised the minority of the infected population. As a result, < 50% of individuals were expected to contribute to non-static transmission. Among the previously identified clustered sequences from this population, approximately 90.6% were considered to be associated with future growth (Fig 6A), indicating the majority of the previously identified clusters are associated with high-risk transmission. As *DeepDynaForecast* considers all leaves for dynamic prediction, regardless of prior cluster assignment, we next sought to evaluate the converse—not surprisingly, < 38% of leaves within the tree predicted by *DeepDynaForecast* to contribute to future growth were previously designated as clustered sequences (Fig 6B). These results indicate the majority of sequences potentially contributing to increased transmission have no clear epidemiological linkage that would result in clustering and would consequently not be considered prioritized individuals using a clustering algorithm alone. An even lower percentage (< 2%) of nodes predicted as declining in transmission rate correspond to previously identified clusters, suggesting transmission cluster identification may not be sufficient for identifying dynamic transmission patterns and high-priority groups for public health interventions.

We next applied a multivariate, logistic regression model to determine if transmission dynamics could be explained by 1) cluster size and 2) available risk factors, regardless of cluster affiliation. Cluster size was significantly associated (*p* < 5 × 10^−8^) with transmission dynamics—individuals within previously identified larger clusters (median of 11 individuals, Supplementary S5 Fig) tended to be predicted as contributing to transmission growth, whereas predicted static and decaying transmission were associated with smaller clusters (median of 5 for both). Importantly, however, decaying clusters contained groups of individuals as large as 37, indicating size alone is not an accurate predictor of transmission dynamics (Supplementary S5 Fig). Turning to the demographic data, despite EHE prioritization based on incidence, EHE county status was highly mixed within the phylogeny (Fig 5) and not considered a significant predictor for transmission dynamics (Fig 6C). Race/ethnicity was, however, considered a significant predictor (*p* < 0.05) for all three dynamic statuses. Gender demonstrated similar association patterns, though was not considered significant. Lastly, as expected, date of birth (DOB) was also considered a significant predictor (*p* < 0.05) for transmission growth and decline, increasing in significance when considered in the context of all dynamic prediction categories in an ordinal regression model (*β* = 0.78, *p* = 0) (Fig 6D). Individuals over 55 years of age (median) were predicted to contribute to slowed transmission, whereas transmission growth was prominent among individuals under the age of approximately 39 years (Fig 6D).

## Discussion

*DeepDynaForecast* is a method based on graph neural network (GNN) principles capable of learning subtle topological patterns within a phylogenetic tree and their association with underlying transmission dynamics. When applied to outbreaks representing both acute and chronic pathogenic infections, *DeepDynaForecast* predicted with 91.6% accuracy the dynamics of transmission clusters within >15, 000 simulated outbreak phylogenies, where 91.9% and 93.0% of lineages contributing to transmission growth and decline, respectively, were identified successfully. Training of the model using stochastic transmission rates and mixed infection types enhanced not only overall performance in comprehensive scenarios but also performance when applied to individual outbreaks separately.

Results of performance validation indicate that *DeepDynaForecast* is a promising tool for transmission characterization in a complex outbreak scenario, though limitations notwithstanding. Validation in this study was restricted to simulated data, which represents not only a limitation to the study but, importantly, a limitation in the reaches of public health. Sequence datasets such as the HIV outbreak in Florida described herein exist in abundance, for which inferences regarding formation of transmission clusters and epidemiologically relevant parameters are made. At the opposite end of the spectrum, contact tracing provides ground truth information regarding transmission chains and detailed information as to rates of secondary infections for individuals within those chains. Integrated studies combining these two data categories—genetic sequences and secondary infections—especially at the level required for outbreak inference, do not exist owing to the extensive workforce and time required for case investigation and tracing. Though an obvious limitation, the lack of existence of these data reinforces the need for models such as *DeepDynaForecast*. Whereas empirical data will always be encouraged, we have demonstrated improved performance of this model achieved with increasing diversity and complexity of the simulations, representing other types of pathogen infection and non-linear transmission dynamics among risk groups.

For sequencing and demographic data collected through molecular surveillance, clustering approaches are commonly used to identify groups at risk of continued transmission, with a focus on larger clusters. Commonly used distance-based clustering approaches, such as HIV-TRACE [8] and MicrobeTrace [33], rely on pre-determined genetic distance thresholds and cannot discriminate groups that represent an increasing risk of transmission from those that show signs of waning transmission. Previous studies have investigated the application of CNNs to genetic distance matrices in predicting infection outbreaks without pre-defined thresholds [10]. However, this approach was concentrated on determining whether individuals are “active” or “inactive” in the context of transmission, falling short of effectively capturing the dynamic changes in populations that can be inferred using phylogenies. Other dynamic forecasting models have been applied specifically to phylogenies and demographic information, which focus only on forecasting growth [9, 14–16] and, again, assume a population structured according to risk groups identified via clustering thresholds. Moreover, growth in these models is defined in terms of number of infected individuals, rather than as a function of transmission rate and so may not capture risk groups for which the transmission rate itself has recently increased, owing to, for example, transient behavioral changes. Alternatively, *DeepDynaForecast* relies solely on the data contained within the given phylogeny (i.e., no thresholds) and focuses on identification of individuals, rather than clusters, that exhibit the potential for future transmission dynamics, inclusive of both growth and decline.

As a result of the differences described above, when applied to an HIV outbreak in the state of Florida from 2012–2017, *DeepDynaForecast* proved capable of corroborating fundamental findings of previous studies but also alluded to a more informative method compared to previous risk group identification through genetic clustering alone. Classification of predicted transmission growth was prevalent among sequences previously identified as belonging to transmission clusters by Rich *et al*. [19]. In particular, the statistical association of predicted transmission growth with larger clusters (>11 individuals) suggests a reasonable rationale for CDC prioritization of larger clusters and is indicative of groups for whom transmission is largely uncontrolled through existing public health approaches. However, as the majority of sequences classified as “growing” according to *DeepDynaForecast* had no clear epidemiological links (i.e., were unclustered), the utility of transmission cluster identification in identifying high-priority groups for public health interventions may be severely limited. An even smaller percentage (< 2%) of nodes classified as decaying, or slowing, transmission were defined as previously clustered individuals, suggesting that interventions may be working for certain populations, but our knowledge of these groups may again be limited by our inability to detect them with existing clustering approaches. It is important to reiterate here that variation may exist between performance of the model on simulated and empirical data, potentially resulting in an unknown degree of false positive dynamic predictions. Additionally, given the reduced accuracy for risk groups comprised of < 20–30 individuals, the potential for misclassification of high-rate, static transmission as growth and low-rate, static transmission as decline is of significance. Lastly, whereas the model was trained on known phylogenetic data, phylogenies reconstructed from genetic sequence data such as in this scenario are considered estimates with varying uncertainty surrounding contextual placement within the tree, which can influence dynamic prediction. These misclassifications should not, however, be discarded so easily. For example, high-rate transmission, even when static over time, is a concern from a public health point of view.

Regardless of the true degree of uncertainty surrounding the Florida HIV outbreak, the associations of age and racial/ethnic group with predicted transmission dynamics within the HIV data are consistent with prior cluster-based prioritization. These findings once again present a message of existence of disproportionate treatment allocation, potentially unrelated to geographical residency within Florida, despite prioritization of specific counties according to HIV incidence as part of the End HIV Epidemic program, as these counties collectively were not considered a significant predictor for transmission dynamics. We elected not to provide the effect ratio among sociodemographic groups because it could be subject by confounding or selection bias, which is not possible to determine within the current framework. We recognize the importance of surveillance data for informing public health policy [35, 36], and we believe that in the principle of beneficence there is a tangible public health benefit in performing research using surveillance data, but we also believe that it is of utmost importance to report results to avoid misinterpretation and risk of harm.

## Methods

### Ethics statement

The authors abide to the Declaration of Helsinki. The study protocol was approved by the University of Florida’s Institutional Review Board (IRB) and by FDOH’s IRB (protocol no. IRB201901041 and 2020–069, respectively) as exempt.

### Simulation of dynamic transmission risk groups

Simulation of early-midway epidemic outbreaks was performed using the *nosoi* [37] agent-based stochastic simulation platform, which is designed to take into account the influence of multiple variables on the transmission process (e.g., population structure and dynamics) to create complex epidemiological simulations. The stochasticity of parameterization further discourages over-fitting of the model to the simulated data. Beginning with a single infected individual, rate of transmission of infection to susceptible individuals varied according to two scenarios—1) transmission of an acute infectious respiratory virus (ARI) or 2) transmission of *Mycobacterium tuberculosis* (TB) among populations of HIV-infected or HIV-uninfected individuals, as described in [38]. Specific paramaters for each outbreak scenario are described in more depth below.

#### Simulation of a structured ARI outbreak

For the simulated ARI outbreak, rate of transmission from an infected individual to a susceptible individual was dependent on the following: 1) the time since initial infection, 2) time duration of infection, 3) number of contacts with susceptible individuals, 4) probability of infection, and 5) the risk group to which the susceptible individual belongs (Supplementary S1 Table). Incubation periods were specified following infection, during which infected individuals were not permitted to transmit (i.e., zero probability of infection transmission). Individuals were removed from the simulation after an infectious period of ∼ 14 days.

Individuals within the simulation were categorized as belonging to the majority, background population (A) or a minority, risk group subpopulation (B-G). Each simulation began with a single infected, background individual and the subsequent infection of another background individual. Following this event, the probability of infection of a background individual was maintained at 1.8 × 10^−3^, relatively higher than that of the individual risk groups. This rate was chosen to ensure risk groups did not exceed the background population in number. The probability of infection was allowed to vary according to risk group, with one stipulation—the probability of infection of dynamic risk groups (growth and decline) was two-fold relative to static groups, owing to their lower representation (2:4, Supplementary S1 Table). Following initiation of infection of a single individual within a risk group, transmission was isolated to the risk group (i.e., probability of zero for infecting an individual in another risk group or background population).

The number of contacts per infected individual varied according to 1) risk group and 2) number of individuals currently infected within that group so as to allow for varying transmission dynamics over time and across risk groups. The number of contacts for groups A-E were picked from a normal distribution with group-specific means and standard deviation, representing groups for which the rate of secondary infection (*R*_*e*_) remained steady, or static. The number of contacts for F and G, however, were derived from the following linear function:

$$\begin{array}{c} N=rh+N_{0}, \end{array}$$ (1)

where *N*_0_ is the initial number of contacts, and *r* is the rate of change dependent on the current number of actively infected hosts in the simulation (*h*). Group F was considered to be experiencing an increasing rate of growth in transmission over time, whereas G was considered to be decaying over time (Supplementary S1 Table). Multiple static transmission risk groups were also incorporated with varying contact parameters in order to determine the ability of the model to distinguish high-rate, static transmission from transmission growth and vice versa. Each of a total of 10, 000 simulations was run for 365 days or until a total of 10, 000 hosts were infected.

#### Simulation of a structured TB/HIV outbreak

A previously described co-infection outbreak model describing the impacts of HIV infection on the spread of TB was adapted herein from Goldstein *et al*. [39]. Briefly, the mean incubation period, though more appropriately referred to as the latent period for TB, was 9 months among hosts that became infectious, and the mean infectious period was 3 months.

Similar to ARI simulations, individuals within the simulation were categorized as belonging to the majority, background population (A) or a minority, risk group subpopulation (B, F, and G). Each simulation also began with a single infected, background individual and the subsequent infection of another background individual. Following this event, the probability of infection of a background individual was 1.8 × 10^−3^. Infection probability was also allowed to vary according to risk group; however, unlike the ARI outbreak, transmission to individuals outside of each risk group was permitted. Two main risk groups were included, representing TB-infected individuals living with or without HIV. These groups were further split into static and dynamic transmitting groups. Based on a reported average of 53% percent of infection recipients harboring HIV [39], infection rate for HIV risk groups A and D were 0.53/2 = 26.5%. Remaining non-HIV-infected risk groups B and E comprised the remaining 47% (equally represented).

Similar to ARI simulations, the number of contacts per infected individual varied according to risk group and number of individuals infected within that group so as to allow for varying transmission dynamics over time and across risk groups. The number of contacts for groups A and B were picked from a normal distribution with group-specific means and standard deviation, representing groups for which the rate of secondary infection (*R*_*e*_) remained steady, or static. Group F was considered to be experiencing an increasing rate of growth in transmission over time, whereas G was considered to be decaying over time (Supplementary S1 Table). Group B was thus considered to exhibit static transmission. Each of a total of 10, 000 simulations was run for 8 years or until a total of 10, 000 hosts were infected.

#### Reconstruction of sampled transmission tree from simulated outbreaks

Recipients were only sampled during their infectious periods, with the sampling time equally likely at any point in this time frame. Only simulations where at least 50 individuals were infected were accepted. Remaining risk groups were sampled randomly, ranging in frequency from 20- 100% of the original risk group population. The background population was also downsampled at a frequency of 20%, representing a more realistic surveillance scenario. Hosts not included within this sample were pruned from the full tree to obtain the final set of simulated trees used for tree statistic calculation and deep learning models. A relaxed molecular clock (evolutionary rate in time) was assumed, and branch lengths were scaled in time (substitutions/site/year) using a uniform distribution rate multiplier ($∼U\left(8\times 10^{-4},0.001\right)$), allowing for both genetic distance and time to be used as distinct weights in the neural network models.

In this study, 10, 000 simulations beginning with a single infected individual were performed on ARI and TB individually, resulting in 8, 574 and 7, 182 successful outbreaks, respectively. In ARI simulations, phylogenetic trees include 33, 713, 775 nodes. Nodes are in three main categories—static, growing, or decaying—based on pre-defined transmission dynamics in the simulation (Supplementary S1 Table), resulting in 31, 920, 116 static, 589, 119 decay and 1, 204, 540 growth samples. For TB simulations, 7, 182 trees include 21, 417, 772 edges and 21, 424, 954 nodes with 20, 884, 036 static, 51, 419 decay, and 489, 499 growth samples. Tree files can be downloaded from the Github repository (https://github.com/lab-smile/DeepDynaForcast).

### Simulation data pre-processing and augmentation

Due to the highly unbalanced input distribution among the three classification categories (see Supplementary S2 and S4 Figs for details), the data represents a realistic scenario in which sampling is biased according to prevalence within the population. Nevertheless, the data was redistributed for model development using a tree-based random split method with a 60%- 20%- 20% ratio. This approach ensures that all nodes of a single phylogenetic tree are only allowed to be split into either the training, validation, or test subset with the proposed probabilities, preventing data leakage. Before feeding the data into the learning algorithms, we performed data pre-processing for raw inputs. For edge features (See Supplementary S2 and S3 Tables), we first applied an inverse hyperbolic sine (ArcSinh) transformation, approximating the natural logarithm of raw values while retaining zero-valued observations, followed by z-score normalization. Due to the difference on the unit of ARI and TB trees, the normalization was performed on each dataset individually. Node features were not available from the simulations but are necessary for *DeepDynaTree* modeling. Therefore, we randomly initialized features of size 16 during the training process to improve model robustness and used fixed zero vectors when testing.

Graph neural network models are limited to emerged nodes in transmission risk groups. To enhance the model’s performance in the early stages of outbreaks with small groups, we utilized a data augmentation strategy for the training data. In each generated group, branches were chopped at a random chopping rate ranging from 0–100%, prioritizing nodes close to the simulation’s end. This downsizes the number of nodes in each cluster, allowing the model to focus on forecasting small group dynamics. The distributions of edge features after pre-processing and data augmentation are summarized in Supplementary S2 and S3 Tables and Supplementary S1 and S3 Figs.

### *DeepDynaForecast* modeling

*DeepDynaForecast* is developed to utilize the topological information and branching patterns within the phylogeny, including genetic distances, to predict the dynamics of external nodes successfully. The phylogenetic tree is modeled as a bi-directed graph, and our model predicts the dynamic status of leaves. The following sections introduce two major components—primal-dual learning structure and cross-layer predictions.

#### Primal-dual graph LSTM module (PDGLSTM)

The PDGLSTM [28] constructs a dual graph (i.e., line graph) of the phylogenetic tree, where the nodes and edges correspond to the tree’s branches and nodes respectively. The PDGLSTM processes both the original (primal) and dual graphs, learning node and edge representations concurrently by applying Graph LSTM (GLSTM) models.

The GLSTM integrates a Long Short-Term Memory (LSTM) model to learn sequential representations that emerge from several message-passing iterations. As depicted in Fig 2, each edge and node aggregates the representations of adjacent nodes or edges. The neural networks *ϕ*_*E*_ and *ϕ*_*N*_ encode these aggregates into low-dimensional messages. These messages are then fed into their corresponding LSTM modules to facilitate the update of node and edge representations.

Formally, given the node representation ${\text{v}}_{i}^{l}$ of node *i* at *l*-th layer and its aggregated message **m**_*i*_, the node representation is updated according to the LSTM updating rule:

$$\begin{array}{c} \begin{array}{cc} & {\text{i}}_{i}^{l}=\sigma \left({\text{W}}_{ii}^{n}{\text{m}}_{i}^{l}+{\text{b}}_{ii}^{n}+{\text{W}}_{hi}^{n}{\text{v}}_{i}^{l}+{\text{b}}_{hi}^{n}\right),\\ & {\text{f}}_{i}^{l}=\sigma \left({\text{W}}_{if}^{n}{\text{m}}_{i}^{l}+{\text{b}}_{if}^{n}+{\text{W}}_{hf}^{n}{\text{v}}_{i}^{l}+{\text{b}}_{hf}^{n}\right),\\ & {\text{g}}_{i}^{l}=\text{tanh}\left({\text{W}}_{ig}^{n}{\text{m}}_{i}^{l}+{\text{b}}_{ig}^{n}+{\text{W}}_{hg}^{n}{\text{v}}_{i}^{l}+{\text{b}}_{hg}^{n}\right),\\ & {\text{o}}_{i}^{l}=\sigma \left({\text{W}}_{io}^{n}{\text{m}}_{i}^{l}+{\text{b}}_{io}^{n}+{\text{W}}_{ho}^{n}{\text{v}}_{i}^{l}+{\text{b}}_{ho}^{n}\right),\\ & c_{i}^{l+1}={\text{f}}_{i}^{l}*c_{i}^{l}+{\text{i}}_{i}^{l}*{\text{g}}_{i}^{l},\\ & {\text{v}}_{i}^{l+1}={\text{o}}_{i}^{l}*\text{tanh}\left(c_{i}^{l+1}\right), \end{array} \end{array}$$ (2)

In these equations, ${\text{W}}_{ix}^{n}$, ${\text{b}}_{ix}^{n}$, ${\text{W}}_{hx}^{n}$ and ${\text{b}}_{hx}^{n}$ are model parameters for *x* ∈ {*i*, *f*, *g*, *o*}. ${\text{i}}_{i}^{l},{\text{f}}_{i}^{l},{\text{g}}_{i}^{l},{\text{o}}_{i}^{l}$ represent the outputs of input gate, forget gate, memory cell and output gate, and *σ*(⋅) denotes sigmoid function. Both **c**_*i*_ and **v**_*i*_ are randomly initialized. Similarly, the edge representation $e_{ij}^{l}$ at layer *l* is updated via an Edge-LSTM model:

$$\begin{array}{c} e_{ij}^{l+1}={\text{LSTM}}_{\text{edge}}\left({\text{m}}_{ij}^{l},e_{ij}^{l}\right), \end{array}$$ (3)

where the **m**_*ij*_ represents the aggregated message for edge *e*_*ij*_ and the detailed computation of *LSTM* is as same as Eq 2, except replacing the node inputs with the edge and its associated message information. In our setting, both Node-LSTM and Edge-LSTM models are configured to have hidden sizes of 128.

Low-dimensional messages are derived by aggregating the representations of adjacent nodes and edges. For example, for node *i*, the aggregated message **m**_*i*_ is created by applying the neural network *ϕ*_*N*_ to the representations of inbound edges and the node itself:

$$\begin{array}{c} {\text{m}}_{i}^{l}=\text{LeakyReLU}({\text{W}}_{N}[{\text{v}}_{i}^{l},\frac{1}{N_{i}}\sum_{j:j\to i}e_{ji}^{l}]+{\text{b}}_{N}), \end{array}$$ (4)

Here, *j* → *i* symbolizes all inbound edges to node *v*_*i*_, and [⋅, ⋅] signifies vector concatenation. **W** and **b** are learnable parameters, mapping the concatenated vector from 2 * 128 = 256 dimensions to a 16-dimensional space.

Similarly, the message information **m**_*ij*_ for edge *e*_*ij*_ is generated by *ϕ*_*E*_:

$$\begin{array}{c} {\text{m}}_{ij}^{l}=\text{LeakyReLU}\left({\text{W}}_{E}\left[{\text{v}}_{i}^{l},e_{ij}^{l},{\text{v}}_{j}^{l}\right]+{\text{b}}_{E}\right), \end{array}$$ (5)

This is achieved by using the representations of edge *e*_*ij*_ and its neighboring nodes *i* and *j*.

#### Cross-layer Prediction module (CLP) and dynamic prediction

Upon completing *L* message-passing iterations, the Cross-layer Prediction (CLP) module predicts the dynamics of leaf nodes while masking the internal nodes during this prediction phase. A series of neural networks, denoted as *ψ*_*L*_, are utilized to predict the leaf node dynamics. These networks use various levels of node representations, **v**. To enhance the robustness of the final result, this process is accompanied by dropout layers and summation operations:

$$\begin{array}{c} {\text{v}}_{i}=\sum_{l=1}^{L}\text{Dropout}({\text{W}}_{L}^{l}{\text{v}}_{i}^{l}+{\text{b}}_{L}^{l}), \end{array}$$ (6)

In this equation, parameters ${\text{W}}_{L}^{l}$ and ${\text{b}}_{L}^{l}$ are associated with the network $\psi_{L}^{l}$ for the node representation at the *l*-th layer. *L* represents the number of message-passing iterations.

The PDGLSTM accumulates extensive information from multi-hop neighboring nodes and edges through iterative application of the primal-dual message passing and state updating steps. This process results in an enhanced, highly informative node representation for the final prediction. In our configuration, the message-passing steps were repeated 30 times. This means the receptive field of each node spans up to 30-hop neighboring nodes and edges. The consolidated node representation, **v**_*i*_, is subsequently processed by a softmax layer to produce the final prediction. The performance of our model with various selections of *L* is detailed in the Supplementary S5 Table. A noticeable performance gap is evident when *L* is set to < 30. For models where *L* > 30, we observed comparable performance levels, albeit at the expense of increased computational costs. Taking both performance and computational efficiency into account, we have chosen *L* = 30 as the optimal setting for our final model. Additionally, this table highlights the significant role of dropout layers in enhancing our model’s predictive accuracy.

### Model evaluation metrics

In this study, our proposed DeepDynaForecast was comprehensively evaluated on six performance metrics, including balanced accuracy, precision, F1-score, area under the receiver operator characteristic curve (AUROC), Brier score (BS), and Cross-entropy (CE). Precision, F1-score, and AUROC are broadly used measures of the discriminability of a pair of classes. To deal with the multi-class case, we first generated the metric values for each class in a one-vs-rest manner and then averaged them with equal weights to give the same importance to each class. Considering the highly unbalanced label distribution in our data set, equally averaging the metrics over all classes instead of weighted averaging by the reference class’s prevalence in the data can effectively avoid overestimating the models that only perform well on the common classes while performing pooling on the rare classes. This equally averaging strategy is also named macro-average. The balanced accuracy is defined as the average of recall obtained on each class [40]. Besides the above metrics calculated based on the “hard” classification results (i.e., final category information only), we also measured the BS and CE to evaluate the models’ “soft” predictions, wherein the metrics were calculated on the prediction probabilities. Eq 7 illustrates the definition of weighted BS and weighted CE values,

$$\begin{array}{c} \begin{array}{cc} BS & =\sum_{n=1}^{N}\frac{1}{K*N_{C_{n}}}\sum_{k=1}^{K}{\left(y_{nk}-{\hat{y}}_{nk}\right)}^{2},\\ CE & =-\sum_{n=1}^{N}\frac{1}{K*N_{C_{n}}}\sum_{k=1}^{K}y_{nk}log\left({\hat{y}}_{nk}\right), \end{array} \end{array}$$ (7)

where ${\hat{y}}_{nk}$ denotes the predicted probability of sample *n* for label *k*, and *y*_*nk*_ is the ground truth with binary value. *C*_*n*_ represents the class of sample *n*. *N* is the number of samples in the data set and *K* is the number of classes where it equals to 3 in our case. Lower BS and CE values thus indicate better prediction performance.

### GCN and GIN baseline models

We selected Graph Convolutional Network (GCN) [29] and Graph Isomorphism Network (GIN) [25] as two baseline models in the dynamic prediction task and trained them on combination of ARI and TB datasets. To ensure a fair comparison, we configured the number of layers to be 20 and set the hidden size at 128. These configurations align with the hyperparameters used in the *DeepDynaForecast*. For GIN, we employed summation as the aggregation type and initiated the *ϵ* value at 0.

### Visualizing node representations with UMAP Projection

We used the Uniform Manifold Approximation and Projection (UMAP) [30] 2D projection to visualize the node representations learned from the *DeepDynaForecast* architecture. We concatenated the hidden representation of nodes from each message-passing iteration into a vector with a size of 20 * 128 = 2560 and selected the top-100 principal components to feed into the UMAP calculation. For the GCN and GIN baseline models, we employed the node representations from just before the last classification layer in the UMAP projection calculations.

### Implementation details

This section introduces the implementation details of *DeepDynaForecast* models. A weighted Cross-entropy loss with inversely proportional to class frequency weights was applied for model optimization. Hyper-parameters of all the methods were extensively optimized on the same validation set for a fair comparison. The model took approximately 1 day to train for around 100 epochs with mini-batch of size 4. An adam optimizer was used for training with an initial learning rate 1 × 10^−3^, and 90% reduction was applied to the learning rate if the validation loss did not improve in the consecutive 50 epoches until reaching the the minimum value 1 × 10^−4^. We finetuned all the hyper-parameters on the validation data set. All experiments were performed on a workstation with 12 Intel Core i7–5930K CPUs and a single Nvidia GeForce GTX TITAN X GPU card.

## Supporting information

S1 Fig Distribution of the edge features on ARI simulations. a, and b, are distributions of the raw edge features: time and genetic distance. c, and d, are distributions of the edge features processed by an ArcSinh transformation and a z-score normalization.
(TIFF)

S2 Fig Label distribution on ARI simulations. a-c are histograms of the number of static, decay, and growth nodes among the trees, and d-f show the distribution of classes’ ratio. Plot g is the histogram of the number of nodes on the trees.
(TIFF)

S3 Fig Distribution of the edge features on TB simulations. a, and b, are distributions of the raw edge features: time and genetic distance. c, and d, are distributions of the edge features processed by an ArcSinh transformation and a z-score normalization.
(TIFF)

S4 Fig Label distribution on TB simulations. a-c are histograms of the number of static, decay, and growth nodes among the trees, and d-f show the distribution of classes’ ratio. Plot g is the histogram of the number of nodes on the trees.
(TIFF)

S5 Fig Cluster sizes for clustered sequences identified as predictive of decaying, static, or growing transmission according to *DeepDynaForecast*. Sequences were clustered previously by Rich *et. al*. [19].
(TIFF)

S1 Table Simulation information for each outbreak and risk group. (PDF)

S2 Table Summary statistics for edge features in ARI. (PDF)

S3 Table Summary statistics for edge features in TB. (PDF)

S4 Table Performance for DDF_ARI_TB. We evaluated the performance of DDF_ARI_TB on different risk groups, including background leaves only (Background leaves), leaves identified as high-risk groups (Risk group leaves), and a combination of them (All leaves). For cluster leaves and all leaves, to mitigate the impact of unbalanced label distribution, metrics—including accuracy, F1-score, precision, and area under the receiver operating characteristic (AUROC)—were uniformly aggregated across the three classes. Weighted Brier score (BS) and weighted Cross-entropy (CE) were calculated based on predicted probabilities adjusted by the inverse prevalence of classes, providing “soft” evaluations of the models. As all background leaves are static, only accuracy, BS, and CE are shown here. For each testing dataset, models were assessed on ARI trees, TB trees, and a combination of both.
(PDF)

S5 Table Performance for DDF_ARI_TB with different recursive depth *L* and ablation study on dropout layers for *L* = 30. (PDF)

S6 Table Performance for DDF with different training data composition. We assessed the performance of the DDF model using six training datasets: half of the ARI dataset (2572 ARI), the entire ARI dataset (5144 ARI), a combination of half of the ARI dataset and an equal number of TB data (2572 ARI + 2572 TB), half of the TB dataset (2154 TB), the full TB dataset (4308 TB), and a mix of half of the TB dataset with an equivalent quantity of ARI data (2154 ARI + 2154 TB). The second column in our results indicates the specific testing dataset used for evaluating the models.
(PDF)
