# Supplementary figures and images for: DeepDynaForecast: Phylogenetic-informed graph deep learning for epidemic transmission dynamic prediction
Source: PLoS Comput Biol. 2024 Apr 10;20(4):e1011351. doi: 10.1371/journal.pcbi.1011351 (PMC11034642; doi:10.1371/journal.pcbi.1011351)

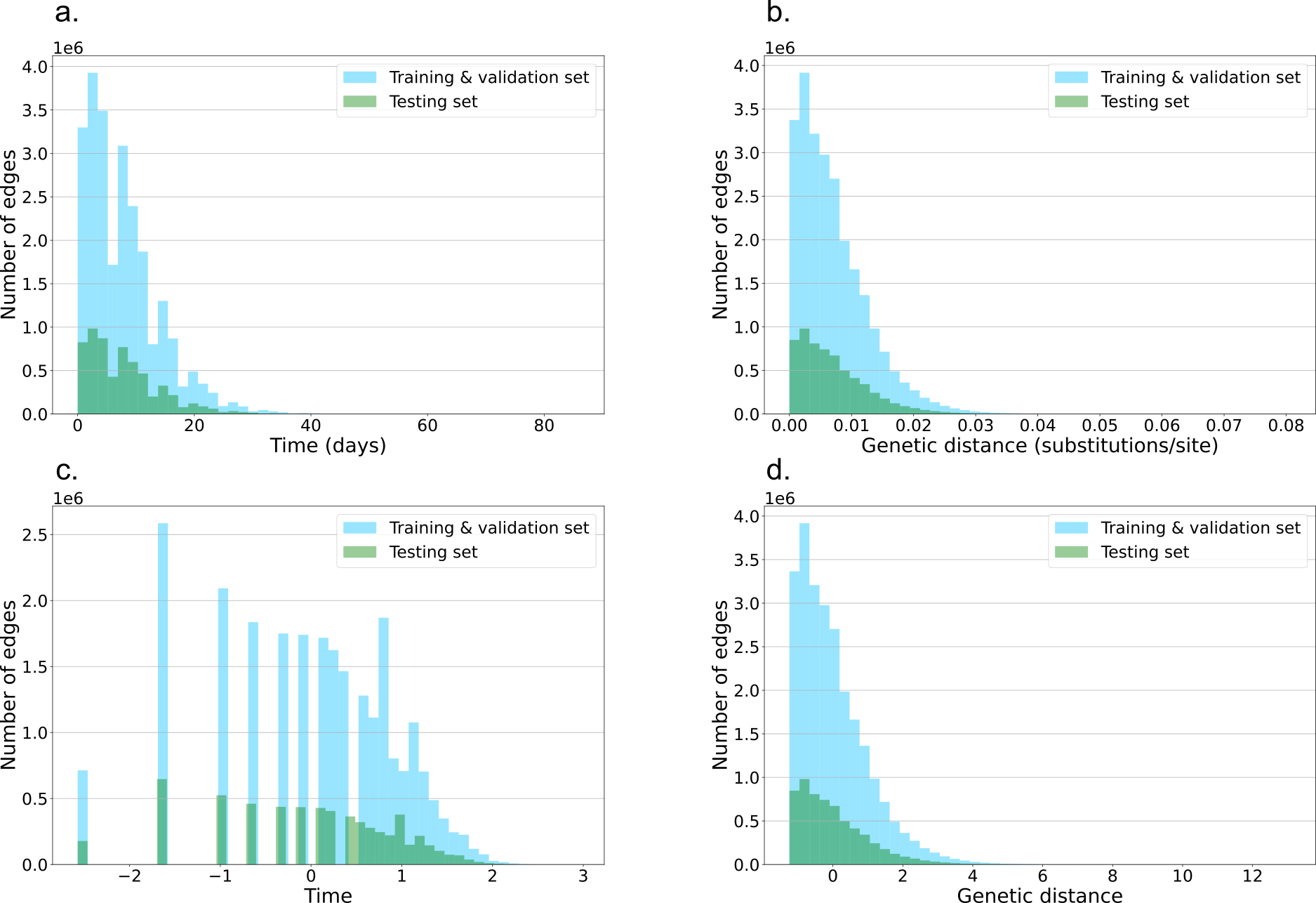

Supplement: S1 Fig — a, and b, are distributions of the raw edge features: time and genetic distance. c, and d, are distributions of the edge features processed by an ArcSinh transformation and a z-score normalization. (TIFF) [file pcbi.1011351.s001.tiff]

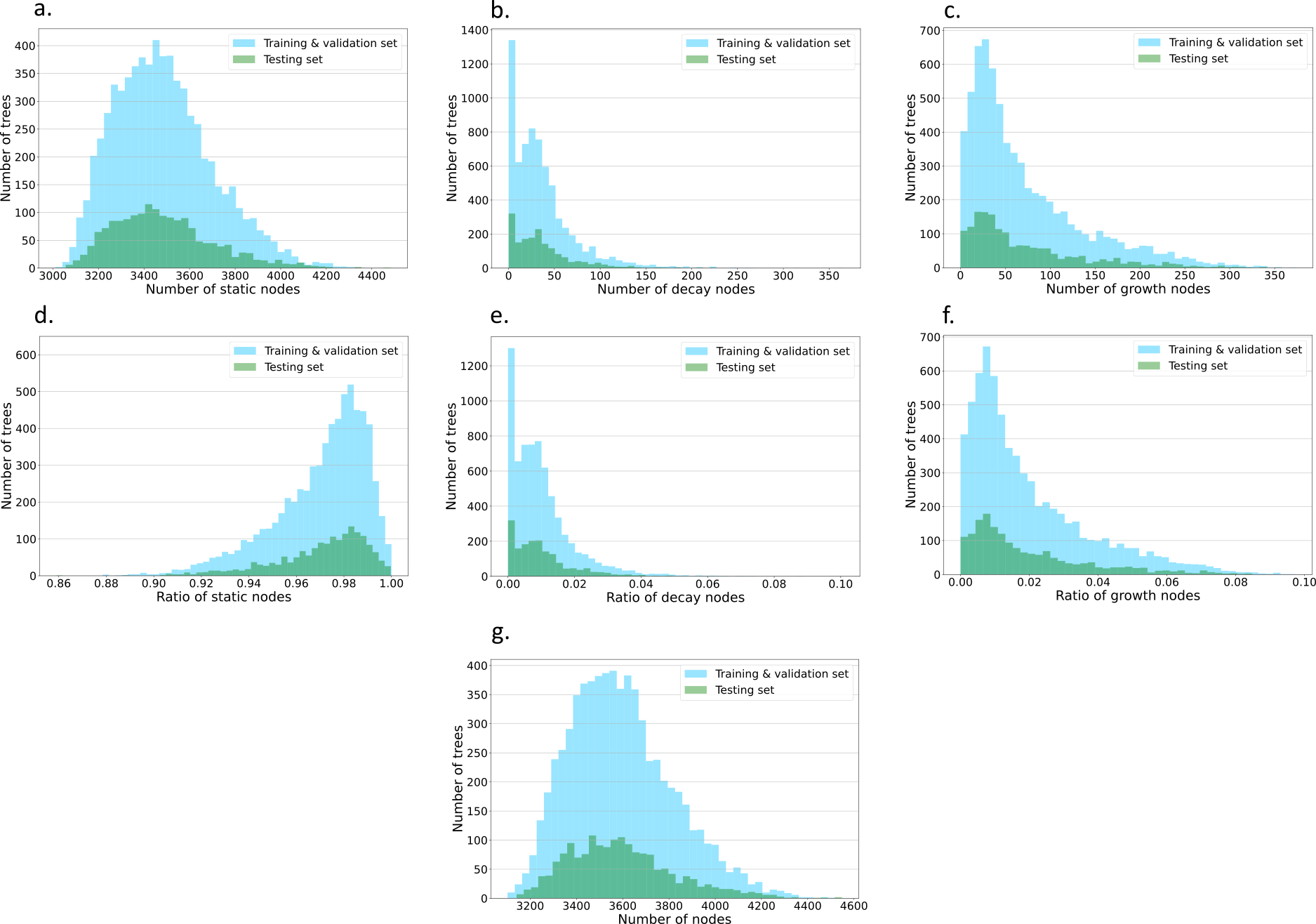

Supplement: S2 Fig — a-c are histograms of the number of static, decay, and growth nodes among the trees, and d-f show the distribution of classes’ ratio. Plot g is the histogram of the number of nodes on the trees. (TIFF) [file pcbi.1011351.s002.tiff]

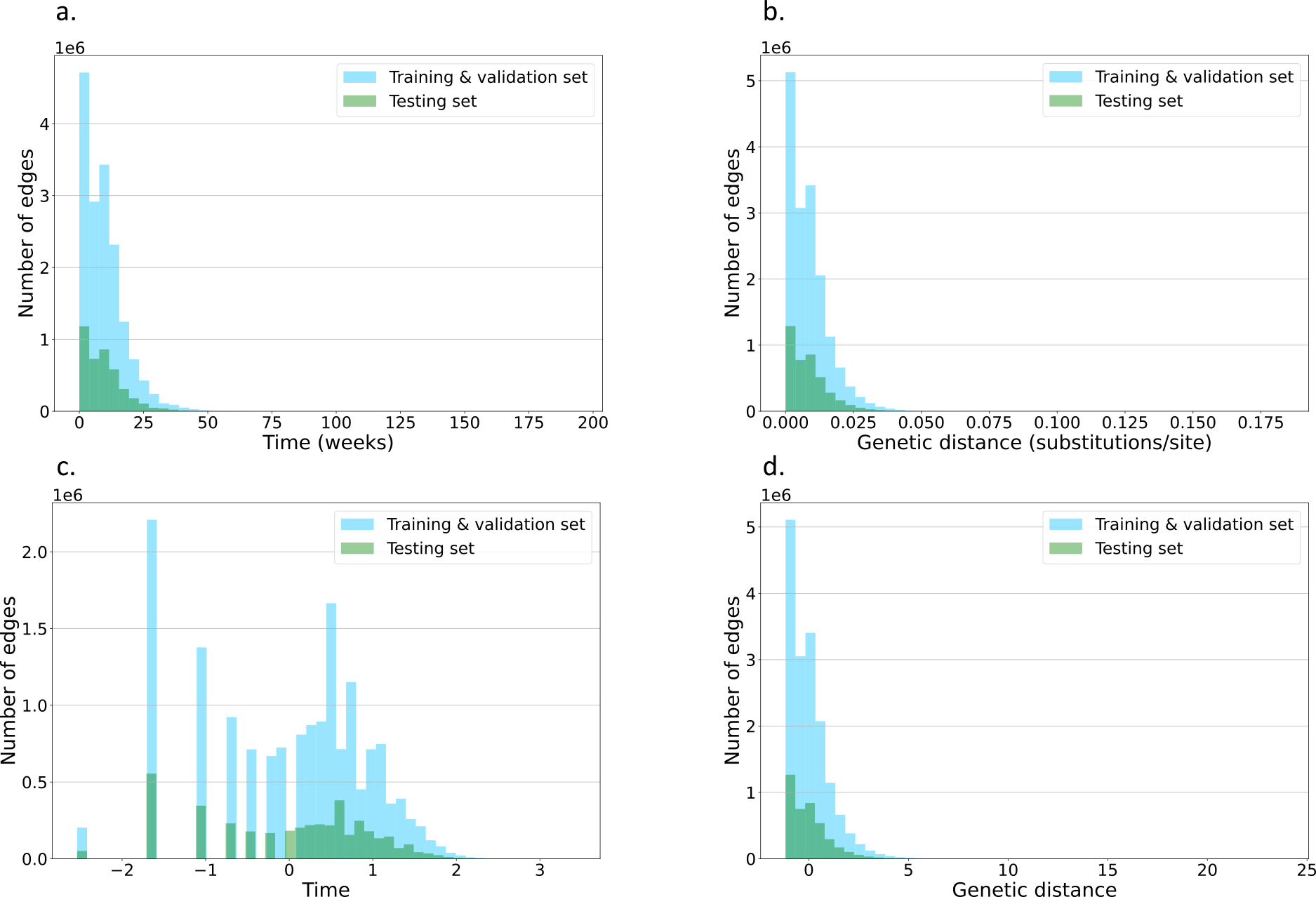

Supplement: S3 Fig — a, and b, are distributions of the raw edge features: time and genetic distance. c, and d, are distributions of the edge features processed by an ArcSinh transformation and a z-score normalization. (TIFF) [file pcbi.1011351.s003.tiff]

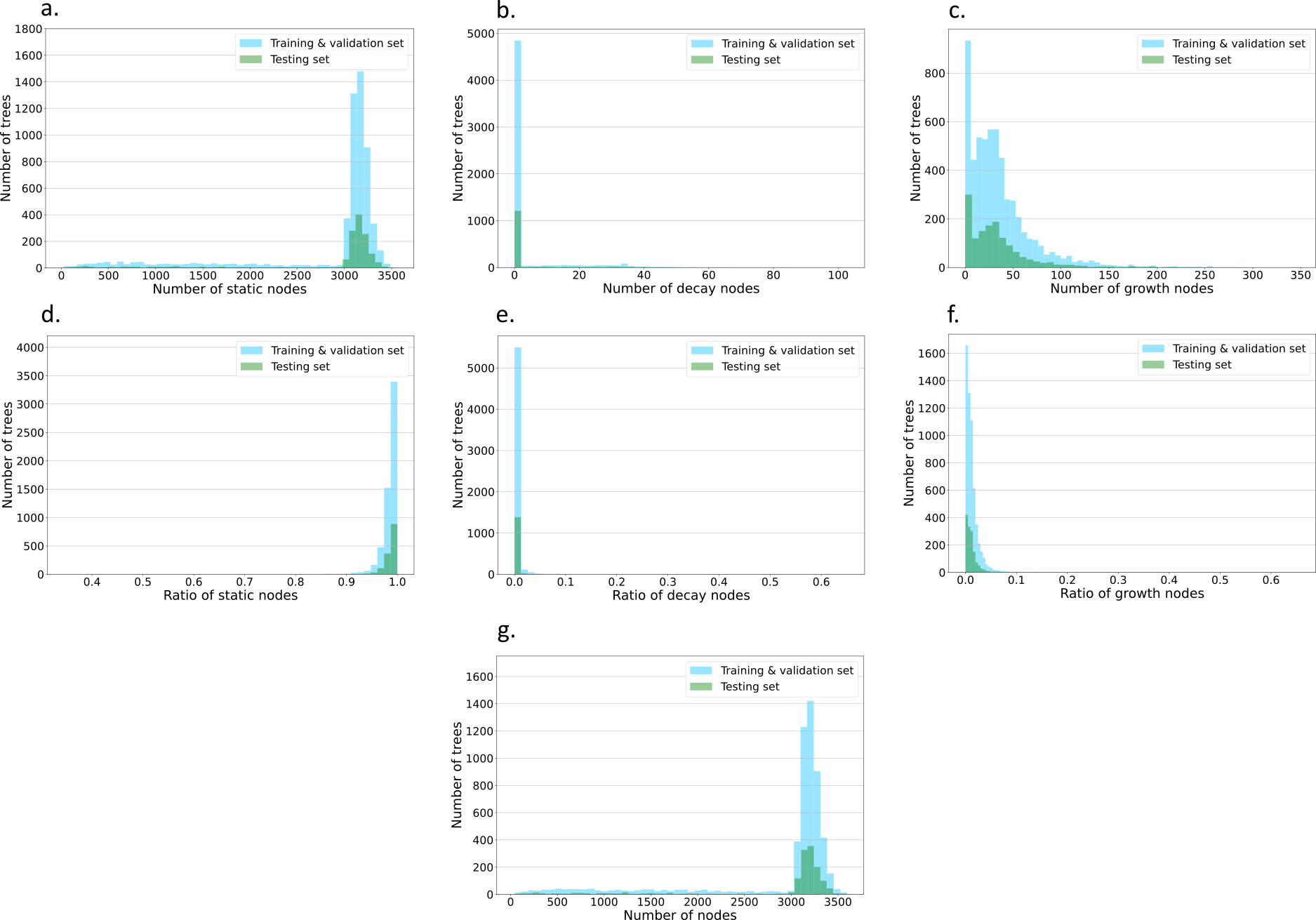

Supplement: S4 Fig — a-c are histograms of the number of static, decay, and growth nodes among the trees, and d-f show the distribution of classes’ ratio. Plot g is the histogram of the number of nodes on the trees. (TIFF) [file pcbi.1011351.s004.tiff]

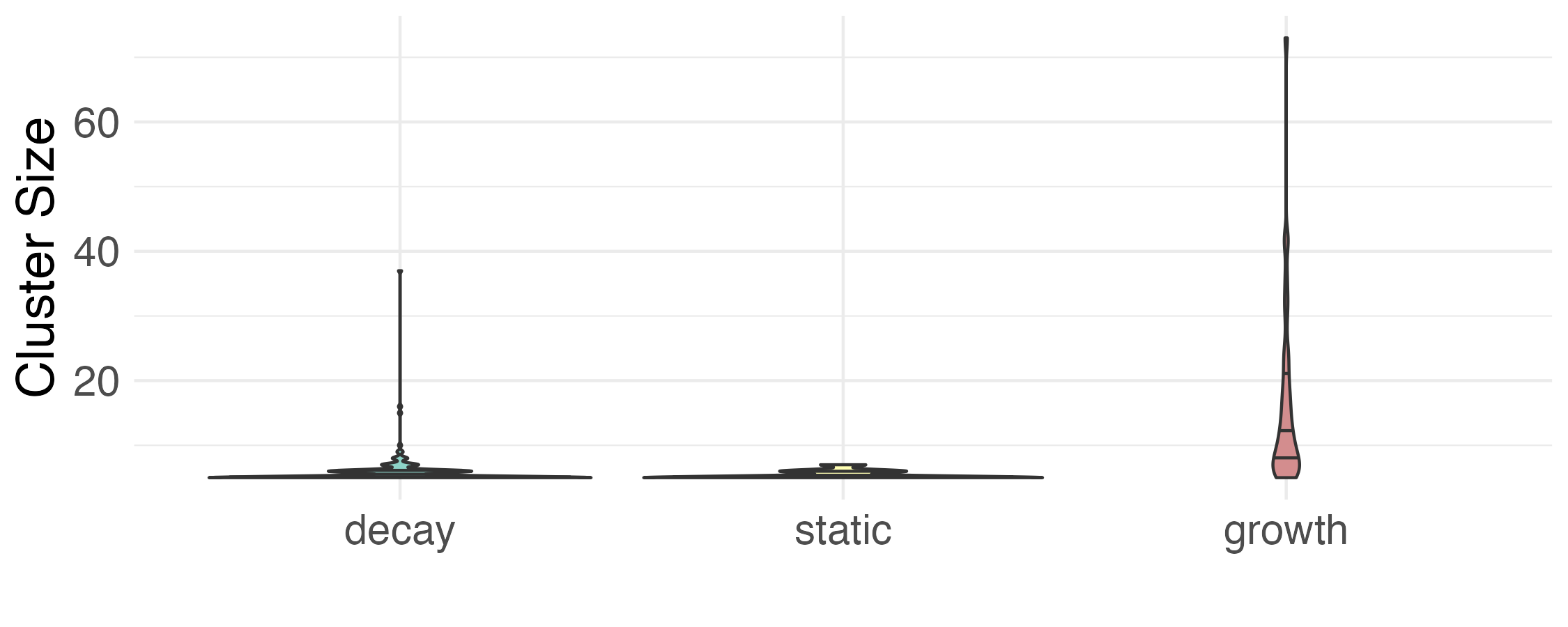

Supplement: S5 Fig — Sequences were clustered previously by Rich et. al. [19]. (TIFF) [file pcbi.1011351.s005.tiff]
