# Supplementary material for: DeepDynaForecast: Phylogenetic-informed graph deep learning for epidemic transmission dynamic prediction
Source: PLoS Comput Biol. 2024 Apr 10;20(4):e1011351. doi: 10.1371/journal.pcbi.1011351 (PMC11034642; doi:10.1371/journal.pcbi.1011351)
Supplement: S1 Table — (PDF) [file pcbi.1011351.s006.pdf]

S1 Table. Simulation information for each outbreak and risk group.

Outbreak scenario	Risk group	Contact parameters[1]	Mean P(T)[2]	Mean R_0
ARI	A	$\sim \mathcal{N}(16, 1)$	0.015	2.2
ARI	B	$\sim \mathcal{N}(4, 1)$	0.09	3.2
ARI	C	$\sim \mathcal{N}(4, 1)$	0.11	3.9
ARI	D	$\sim \mathcal{N}(6, 1)$	0.09	4.9
ARI	E	$\sim \mathcal{N}(4, 1)$	0.12	4.3
ARI	F	$N_0 = \sim \mathcal{N}(4,\ 1)$, $r = \sim \mathcal{N}(0.018,\ 0.009)$	0.11	0.8
ARI	G	$N_0 = \sim \mathcal{N}(6,\ 1)$, $r = \sim \mathcal{N}(0.18,\ 0.09)$	0.11	9.8
TB/HIV	A	$\sim \mathcal{N}(20, 5)$	0.025	1.5
TB/HIV	B	$\sim \mathcal{N}(20, 5)$	0.015	0.9
TB/HIV	F	$N_0 = \sim \mathcal{N}(15,\ 1)$, $r = \sim \mathcal{N}(0.18,\ 0.09)$	0.015	0.7
TB/HIV	G	$N_0 = \sim \mathcal{N}(20,\ 1)$, $r = \sim \mathcal{N}(1.8,\ 0.9)$	0.025	1.5

[1] Single values represent mean number of contacts for static transmission groups.
[2] Probability of transmission
