# Supplementary material for: DeepDynaForecast: Phylogenetic-informed graph deep learning for epidemic transmission dynamic prediction
Source: PLoS Comput Biol. 2024 Apr 10;20(4):e1011351. doi: 10.1371/journal.pcbi.1011351 (PMC11034642; doi:10.1371/journal.pcbi.1011351)
Supplement: S2 Table — (PDF) [file pcbi.1011351.s007.pdf]

S2 Table. Summary statistics for edge features in ARI.

**Features**	**Min**	**Max**	**Mean**	**Std**
Time (days)	0.000	8.600×10^{1}	7.771	6.221
Genetic distance (substitutions/site)	0.000	8.072×10^{-2}	6.994×10^{-3}	5.629×10^{-3}
