# Supplementary material for: DeepDynaForecast: Phylogenetic-informed graph deep learning for epidemic transmission dynamic prediction
Source: PLoS Comput Biol. 2024 Apr 10;20(4):e1011351. doi: 10.1371/journal.pcbi.1011351 (PMC11034642; doi:10.1371/journal.pcbi.1011351)
Supplement: S3 Table — (PDF) [file pcbi.1011351.s008.pdf]

S3 Table. Summary statistics for edge features in TB.

**Features**	**Min**	**Max**	**Mean**	**Std**
Time (months)	0.000	1.940×10^{2}	9.470	8.067
Genetic distance (substitutions/site)	0.000	1.834×10^{-1}	8.524×10^{-3}	7.296×10^{-3}
