# Supplementary material for: DeepDynaForecast: Phylogenetic-informed graph deep learning for epidemic transmission dynamic prediction
Source: PLoS Comput Biol. 2024 Apr 10;20(4):e1011351. doi: 10.1371/journal.pcbi.1011351 (PMC11034642; doi:10.1371/journal.pcbi.1011351)
Supplement: S4 Table — We evaluated the performance of DDF_ARI_TB on different risk groups, including background leaves only (Background leaves), leaves identified as high-risk groups (Risk group leaves), and a combination of them (All leaves). For cluster leaves and all leaves, to mitigate the impact of unbalanced label distribution, metrics—including accuracy, F1-score, precision, and area under the receiver operating characteristic (AUROC)—were uniformly aggregated across the three classes. Weighted Brier score (BS) and weighted Cross-entropy (CE) were calculated based on predicted probabilities adjusted by the inverse prevalence of classes, providing “soft” evaluations of the models. As all background leaves are static, only accuracy, BS, and CE are shown here. For each testing dataset, models were assessed on ARI trees, TB trees, and a combination of both. (PDF) [file pcbi.1011351.s009.pdf]

S4 Table. Performance for DDF_ARI_TB.

Dataset	Leaf group	Accuracy ↑	F1 ↑	Precision ↑	AUROC ↑	BS ↓	CE ↓
ARI	Background leaves	0.932	–	–	–	0.102	0.185
	Risk group leaves	0.740	0.584	0.653	0.887	0.425	0.742
	All leaves	0.931	0.545	0.477	0.982	0.108	0.200
TB	Background leaves	0.935	–	–	–	0.108	0.216
	Risk group leaves	0.649	0.439	0.476	0.830	0.499	0.849
	All leaves	0.816	0.433	0.401	0.954	0.271	0.492
ARI+TB	Background leaves	0.933	–	–	–	0.104	0.198
	Risk group leaves	0.731	0.556	0.599	0.876	0.422	0.728
	All leaves	0.916	0.512	0.452	0.977	0.128	0.237

We evaluated the performance of DDF_ARI_TB on different risk groups, including background leaves only (Background leaves), leaves identified as high-risk groups (Risk group leaves), and a combination of them (All leaves). For cluster leaves and all leaves, to mitigate the impact of unbalanced label distribution, metrics—including accuracy, F1-score, precision, and area under the receiver operating characteristic (AUROC)—were uniformly aggregated across the three classes. Weighted Brier score (BS) and weighted Cross-entropy (CE) were calculated based on predicted probabilities adjusted by the inverse prevalence of classes, providing "soft" evaluations of the models. As all background leaves are static, only accuracy, BS, and CE are shown here. For each testing dataset, models were assessed on ARI trees, TB trees, and a combination of both.
