# Supplementary material for: DeepDynaForecast: Phylogenetic-informed graph deep learning for epidemic transmission dynamic prediction
Source: PLoS Comput Biol. 2024 Apr 10;20(4):e1011351. doi: 10.1371/journal.pcbi.1011351 (PMC11034642; doi:10.1371/journal.pcbi.1011351)
Supplement: S5 Table — (PDF) [file pcbi.1011351.s010.pdf]

**S5 Table. Performance for DDF_ARI_TB with different recursive depth $L$ and ablation study on dropout layers for $L = 30$.**

| Model setting | Accuracy ↑ | F1 ↑ | Precision ↑ | AUROC ↑ | BS ↓ | CE ↓ |
|---|---|---|---|---|---|---|
| L=1 | 0.333 | 0.012 | 0.006 | 0.500 | 0.671 | 1.105 |
| L=2 | 0.481 | 0.242 | 0.344 | 0.673 | 0.592 | 0.981 |
| L=5 | 0.575 | 0.307 | 0.356 | 0.750 | 0.509 | 0.841 |
| L=10 | 0.708 | 0.362 | 0.379 | 0.858 | 0.387 | 0.650 |
| L=20 | 0.881 | 0.459 | 0.424 | 0.964 | 0.175 | 0.315 |
| L=30 | **0.916** | **0.512** | **0.452** | **0.977** | **0.128** | **0.237** |
| L=30 w/o Dropout | 0.904 | 0.491 | 0.440 | 0.961 | 0.143 | 0.262 |
| L=40 | 0.910 | 0.509 | 0.450 | 0.974 | 0.137 | 0.253 |
| L=50 | 0.913 | 0.507 | 0.450 | 0.975 | 0.132 | 0.244 |
