# Supplementary material for: DeepDynaForecast: Phylogenetic-informed graph deep learning for epidemic transmission dynamic prediction
Source: PLoS Comput Biol. 2024 Apr 10;20(4):e1011351. doi: 10.1371/journal.pcbi.1011351 (PMC11034642; doi:10.1371/journal.pcbi.1011351)
Supplement: S6 Table — We assessed the performance of the DDF model using six training datasets: half of the ARI dataset (2572 ARI), the entire ARI dataset (5144 ARI), a combination of half of the ARI dataset and an equal number of TB data (2572 ARI + 2572 TB), half of the TB dataset (2154 TB), the full TB dataset (4308 TB), and a mix of half of the TB dataset with an equivalent quantity of ARI data (2154 ARI + 2154 TB). The second column in our results indicates the specific testing dataset used for evaluating the models. (PDF) [file pcbi.1011351.s011.pdf]

S6 Table. Performance for DDF with different training data composition.

Training data	Testing data	Accuracy ↑	F1 ↑	Precision ↑	AUROC ↑	BS ↓	CE ↓
2572 ARI	ARI	0.881	0.479	0.435	0.962	0.182	0.343
5144 ARI	ARI	0.909	0.539	0.473	0.975	0.140	0.264
2572 ARI + 2572 TB	ARI	0.910	0.534	0.468	0.973	0.139	0.259
2154 TB	TB	0.749	0.441	0.406	0.941	0.366	0.653
4308 TB	TB	0.768	0.461	0.419	0.950	0.339	0.608
2154 ARI + 2154 TB	TB	0.756	0.407	0.388	0.923	0.343	0.603

We assessed the performance of the DDF model using six training datasets: half of the ARI dataset (2572 ARI), the entire ARI dataset (5144 ARI), a combination of half of the ARI dataset and an equal number of TB data (2572 ARI + 2572 TB), half of the TB dataset (2154 TB), the full TB dataset (4308 TB), and a mix of half of the TB dataset with an equivalent quantity of ARI data (2154 ARI + 2154 TB). The second column in our results indicates the specific testing dataset used for evaluating the models.
